# The Influence of Physical Activity and Diet Mobile Apps on Cardiovascular Disease Risk Factors: Meta-Review

**DOI:** 10.2196/51321

**Published:** 2024-10-09

**Authors:** Erica Bushey, Yin Wu, Alexander Wright, Linda Pescatello

**Affiliations:** 1 University of Connecticut Storrs, CT United States; 2 Hartford Hospital Hartford, CT United States

**Keywords:** physical activity, diet, mobile applications, obesity, hypertension, dyslipidemia, diabetes, mobile phone

## Abstract

**Background:**

The literature on whether physical activity (PA) and PA and diet (PA+Diet) mobile apps improve cardiovascular disease (CVD) risk factors is promising.

**Objective:**

The aim of this meta-review is to provide an evidence synthesis of systematic reviews and meta-analyses examining the influence of PA and PA+Diet apps on the major CVD risk factors.

**Methods:**

We systematically searched 5 databases until January 12, 2022. Included systematic reviews and meta-analyses (1) reported the CVD risk factor outcomes of BMI, waist circumference, body weight, blood pressure (BP), hemoglobin A_1c_ (HbA_1c_), fasting blood glucose, blood lipids, or PA; (2) enrolled healthy participants ≥18 years who may or may not have the metabolic syndrome, diabetes mellitus, or preexisting CVD risk factors; (3) reviewed PA or PA+Diet app interventions integrating behavioral change techniques (BCT) to deliver their information; and (4) had a nonapp control.

**Results:**

In total, 17 reviews (9 systematic reviews and 8 meta-analyses) published between 2012 and 2021 qualified. Participants were middle-aged, mostly women ranging in number from 10 to 62,219. Interventions lasted from 1 to 24 months, with the most common behavioral strategies being personalized feedback (n=8), self-monitoring (n=7), and goal setting (n=5). Of the PA app systematic reviews (N=4), the following CVD risk factors improved: body weight and BMI (n=2, 50%), BP (n=1, 25%), HbA_1c_ (n=1, 25%), and blood lipids (n=1, 25%) decreased, while PA (n=4, 100%) increased. Of the PA+Diet app systematic reviews (N=5), the following CVD risk factors improved: body weight and BMI (n=3, 60%), BP (n=1, 20%), and HbA_1c_ (n=3, 60%) decreased, while PA (n=3, 60%) increased. Of the PA app meta-analyses (N=1), the following CVD risk factors improved: body weight decreased (–0.73 kg, 95% CI –1.45 to –0.01; *P*=.05) and PA increased by 25 minutes/week (95% CI 0.58-1.68; *P*<.001), while BMI (–0.09 kg/m^2^, 95% CI –0.29 to 0.10; *P*=.35) and waist circumference (–1.92 cm, 95% CI –3.94 to 0.09; *P*=.06) tended to decrease. Of the PA+Diet app meta-analyses (n=4), the following CVD risk factors improved: body weight (n=4, 100%; from –1.79 kg 95% CI –3.17 to –0.41; *P*=.01 to –2.80 kg 95% CI –4.54 to –1.06, *P*=.002), BMI (n=1, 25%; –0.64 kg/m^2^, 95% CI –1.09 to –0.18; *P*=.01), waist circumference (n=1, 25%; –2.46 cm, 95% CI –4.56 to –0.36; *P*=.02), systolic/diastolic BP (n=1, 25%; –4.22/–2.87 mm Hg, 95% CI –6.54 to –1.91/ –4.44 to –1.29; *P*<.01), and HbA_1c_ (n=1, 25%; –0.43%, 95% CI –0.68 to –0.19; *P*<.001) decreased.

**Conclusions:**

PA and PA+Diet apps appear to be most consistent in improving PA and anthropometric measures with favorable but less consistent effects on other CVD risk factors. Future studies are needed that directly compare and better quantify the effects of PA and PA+Diet apps on CVD risk factors.

**Trial Registration:**

PROSPERO CRD42023392359; https://www.crd.york.ac.uk/prospero/display_record.php?RecordID=392359

## Introduction

### Background

Cardiovascular Disease (CVD) is the leading cause of death in the United States and the world [1], with an estimated 1 in 3 adults dying from CVD [2]. Between 2018 and 2019, direct and indirect costs of CVD were US $407.3 billion, making it the costliest disease in the United States [3]. Identifying those at risk for CVD is important due to the significant socioeconomic burden CVD imposes [1]. The major CVD risk factors are physical inactivity, obesity, diabetes mellitus (DM), dyslipidemia, and hypertension, with a prevalence ranging from 11% for DM to 75% for physical inactivity among US adults [1,2,4-8]. Half of US adults have 1 or more CVD risk factors [4-8]. Professional guidelines recommend lifestyle modifications, notably physical activity (PA) and diet, as critical first steps to prevent and treat CVD and its risk factors [2,9-11].

Mobile health apps have proliferated because of their accessibility, affordability, increased smartphone usage, and improved technology [12]. The global health care mobile app market size was valued at US $17.92 billion in 2019 and is expected to grow at a mean annual growth rate of 45% from 2020 to 2027 [12]. PA and Diet (PA+Diet) apps dominate the health App market with a share of 54.8% in 2021 [13]. Indeed, the market size of fitness and health apps is expected to increase globally by 46% from US $17.92 billion between 2020 and 2027 [13]. Evidence is growing that PA and PA+Diet apps have key functions that favorably impact CVD risk factors such as PA and hypertension [9-11,14-19]. These features include enabling patient education and selfcare through the use of self-monitoring and self-management, remote monitoring of patients with clinician online care encounters through digital platforms, and research to determine intervention effectiveness [14].

Due to the popularity of PA and PA+Diet apps, the number of systematic reviews, meta-analyses, and randomized controlled trials (RCT) investigating the influence of these apps on CVD risk factors has increased substantially [20-26]. Indeed, we conducted searches in PubMed with terms related to the various types of reviews on January 11, 2023, and found 1011 “systematic reviews, meta-analyses, and RCTs involving PA apps” from 2010 to 2023; and 268 “systematic reviews, meta-analyses, and RCTs involving PA+Diet apps” from 2009 to 2023 [20]. A meta-review is a growing type of evidence synthesis that gathers systematic reviews and meta-analyses and evaluates and summarizes the research questions being examined [27]. Surprisingly, to the best of our knowledge, there are no meta-reviews evaluating the impact of PA and PA+Diet apps on CVD risk factors.

### Purpose

We performed this meta-review to provide the first evidence synthesis of systematic reviews and meta-analyses examining the influence of PA and PA+Diet apps on the major CVD risk factors of physical inactivity, obesity, DM, dyslipidemia, and hypertension.

## Methods

### Search Strategy

**Figure 1:**
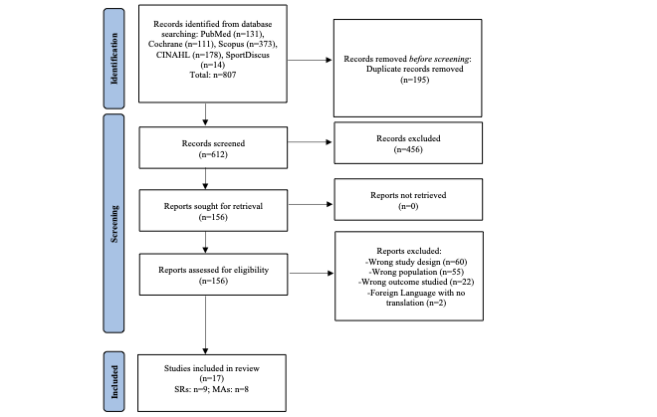
PRISMA (Preferred Reporting Items for Systematic Reviews and Meta-Analyses) search strategy.

This meta-review is reported consistent with the PRISMA (Preferred Reporting Items for Systematic Reviews and Meta-Analyses) statement [28,29]. The PRISMA diagram of the search strategy is found in Figure 1. With the assistance of a medical librarian, we systematically searched 5 databases (PubMed, Scopus, SportDiscus, Cumulative Index to Nursing and Health Literature, and Cochrane Library) from inception to January 12, 2022 (see Multimedia Appendix 1 for the detailed list of search terms). The protocol is registered in PROSPERO (CRD42023392359).

To be classified as an app, the app integrated a software program designed to run on a mobile device such as a phone or tablet that reported CVD risk factor outcomes [13]. A PA intervention within a PA app included strategies to increase PA that ranged from prescribed exercise programs, reminders to exercise, or recommendations to meet the PA guidelines of 150 to 300 minutes/week of moderate-intensity or 75 to 150 minutes/week of vigorous-intensity aerobic PA or some combination of both [6,30]. A diet intervention within a PA+Diet app included strategies to improve eating behaviors that range from keeping daily food logs, nutritional counseling, and meeting the dietary guidelines of a healthy dietary pattern to meet nutrient needs, help achieve a healthy body weight, and reduce the risk of chronic disease [5,31]. PA and PA+Diet app interventions also included behavioral change techniques (BCT) (ie, goal setting and social support) to deliver their intervention [12].

Included systematic reviews and meta-analysis of PA and PA+Diet apps: (1) reported the CVD risk factor outcomes of anthropometrics (ie, BMI, waist circumference, and body weight); blood pressure (BP; ie, systolic blood pressure [SBP] and diastolic blood pressure [DBP]); glycemic biomarkers (ie, fasting blood glucose, hemoglobin A_1c_ [HbA_1c_]), blood lipid-lipoproteins (ie, total cholesterol, triglycerides, high-density lipoprotein [HDL], and low-density lipoprotein [LDL]), or PA volume (ie, minutes/week, steps/day); (2) enrolled healthy individuals ≥18 years who may or may not have metabolic syndrome, diabetes mellitus, or preexisting CVD risk factors; (3) reviewed PA or PA+Diet app interventions integrating BCTs to deliver their information; and (4) had a non-app control that consisted of usual care or a waitlist.

### Data Extraction and Coding

A systematic review screening tool, Rayyan, was used to triage the 807 potentially qualifying studies [32]. After duplicates were removed, the 612 potentially relevant remaining reports were screened first by title, abstract, and full text (more details in Figure 1). For the 17 qualifying reports (n=9 systematic reviews and n=8 meta-analyses), data were extracted related to the study characteristics (eg, publication year), sample characteristics (eg, gender, age), intervention characteristics (eg, app features), and outcomes (eg, BMI, waist circumference, BP). Reference lists of all included studies were manually searched and cross-referenced for additional reports. Data were extracted from the qualifying studies using a standardized coding form and coding manual previously developed and used by members of our research team [33]. A total of 2 trained coders (EB, AW) independently extracted and coded study information. All disagreements were resolved through discussion (with a third investigator if necessary). The outcomes of the PA and PA+Diet app systematic reviews (n=9) and meta-analyses (n=8) are presented in Table 1. For the systematic reviews, CVD risk factor outcomes were coded as either the CVD risk factor outcomes favored PA or PA+Diet apps over control, or there was no difference in CVD risk factor outcomes between PA or PA+Diet apps over control. For the MAs, CVD risk factor outcomes were coded by the standardized and unstandardized effect sizes. We categorized the effect size as (1) Favors PA or PA+Diet apps when the effect size was negative (or positive for HDL and PA) and statistically significant, (2) Favors Control when the effect size was positive (or negative for HDL and PA) and statistically significant, and (3) No Difference between PA or PA+Diet apps vs control when the effect size was not statistically significant.

**Table 1:** Physical activity, and physical activity and diet app systematic reviews (n=9) and meta-analyses (n=8) cardiovascular disease risk factor outcomes.

Outcome	PA^a^ apps		PA and diet apps	
	Systematic reviews (n=4)	Meta-analyses (n=1)	Systematic reviews (n=5)	Meta-analyses (n=7)
BMI	—^b^	X^c^	X	X
Body weight	X	X	X	X
Waist circumference	—	—	—	X
Blood pressure	X	—	X	X
Hemoglobin A_1c_	X	—	X	X
Fasting blood glucose	—	—	—	X
Total cholesterol	X	—	—	—
Triglycerides	—	—	—	—
High-density lipoprotein	—	—	—	X
Low-density lipoprotein	X	—	—	—
Physical activity	X	X	X	X

^a^PA: physical activity.

^b^Not available.

^c^X indicates which outcomes were assessed.

### Study Methodological Quality Assessment

Study methodological quality was assessed by 2 trained coders (EB and AW) independently using A Measurement Tool for Assessment of Multiple Systematic Reviews (AMSTAR) checklist containing 18 items [34]. Systematic reviews meeting <9 (50%) of the items were rated as low, those meeting between 9 and <13 (50%-69%) of the items were rated as moderate, and those meeting >13 (>70%) were rated as high study methodological quality. All disagreements were resolved through discussion (with a third investigator if necessary). See Multimedia Appendix 2 for a summary of the methodological quality assessment from the AMSTAR Checklist.

## Results

The PRISMA diagram of the search strategy is found in Figure 1 (checklist in Multimedia Appendix 3). We identified 807 potentially qualifying systematic reviews and meta-analyses. Only 1 primary-level study was included in 4 of the identified reviews, so the overlap in primary studies across the included reviews was minimal [35]. Out of these, we excluded 195 duplicates, 456 articles based on title and abstract, and 156 articles on full-text review. We identified 17 reports (9 systematic reviews and 8 meta-analyses) involving 269 studies satisfying the inclusion criteria. Of the 9 systematic reviews, 4 evaluated PA apps [36-39], and 5 evaluated PA+Diet apps [40-44]. Out of the 8 meta-analyses, 1 evaluated PA apps [45], and 7 evaluated PA+Diet apps [46-52].

### Study Characteristics

Multimedia Appendix 4 contains the descriptions of the sample, study, and intervention characteristics of the qualifying systematic reviews or meta-analyses. The systematic reviews (n=9) and meta-analyses (n=8) involving PA and PA+Diet apps were published between 2012 and 2021 with most research groups located in the United States (5/17, 29%) and United Kingdom (5/17, 29%) followed by Spain (3/17, 18%), Taiwan (1/17, 6%), Canada (1/17, 6%), Korea (1/17, 6%), and Australia (1/17, 6%). The systematic reviews (n=4) and meta-analysis (n=1) involving PA apps only were published between 2018 and 2021, with most research groups located in the United States (2/5, 40%) followed by Canada (1/5, 20%), the United Kingdom (1/5, 20%), and Australia (1/5, 20%). The systematic reviews (n=5) and meta-analysis (n=7) involving PA+Diet apps were published between 2012 and 2021, with most research groups located in the United Kingdom (4/12, 33%), Spain (3/12, 25%), and the United States (3/12, 25%) followed by Taiwan (1/12, 8%), and Korea (1/12, 8%).

### Sample Characteristics

The systematic reviews (n=9) and meta-analyses (n=8) involving PA and PA+Diet apps had participants ranging in number from 10 to 62,219 [37,42,45-47,50] who were mostly middle-aged women ranging in age from 18 to 67 years [37,42,45-47,50]. No qualifying systematic reviews and meta-analyses involving PA and PA+Diet apps disclosed the ethnicity or race or the baseline CVD risk factors values of the study participants. The systematic reviews (n=4) and meta-analysis (n=1) involving PA apps only (n=5) had participants ranging in age from 10 to 69, 219 who were mostly middle-aged women ranging in age from 18 to 65 years. The systematic reviews (n=5) and meta-analysis (n=7) involving PA+Diet apps (n=12) had participants ranging in number from 10 to 1,386, who were mostly middle-aged women ranging in age from 18 to 67 years. The systematic reviews (n=2) and meta-analysis (n=8) involving PA and PA+Diet apps conducted risk of bias assessments, 8 of which used the Cochrane Collaboration tool for assessing the risk of bias in randomized trials. Overall, the risk of bias between the 10 reviews was low to moderate.

### Intervention Characteristics

The systematic reviews (n=9) and meta-analyses (n=8) involving PA and PA+Diet apps had interventions that lasted between 1 and 24 months, with the most common durations being 6 weeks (3/17, 18%) and 8 weeks (3/17, 18%). The systematic reviews (n=4) and meta-analyses (n=1) involving PA apps only lasted between 6 weeks to 12 months, with the most common duration being 6 weeks (2/5, 40%). The systematic reviews (n=5) and meta-analyses (n=7) involving PA+Diet apps lasted between 4 weeks to 24 months, with the most common duration being 8 weeks (3/12, 25%). The systematic reviews (n=9) and meta-analyses (n=8) involving PA and PA+Diet apps used personalized feedback (8/17, 47%), self-monitoring (7/17, 41%), and goal setting (5/17, 29%) as the most common behavioral strategies. Other behavioral strategies included education (2/17, 12%), PA recommendations (1/17, 6%), reminders (1/17, 6%), and social support (1/17, 6%). The systematic reviews (n=4) and meta-analyses (n=1) involving PA apps only used personalized feedback (1/5, 20%), goal setting (1/5, 20%), and PA reminders (1/5, 20%) as the most common behavioral strategies. The systematic reviews (n=5) and meta-analyses (n=7) involving PA+Diet apps used personalized feedback (7/12, 58%), self-monitoring (7/12, 58%), and goal setting (4/12, 33%) as the most common behavioral strategies. The systematic reviews (n=9) and meta-analyses (n=8) involving PA and PA+Diet apps used either web-based applications (12/17, 71%) or mobile phone apps (11/17, 65%) as their platform. The systematic reviews (n=4) and meta-analyses (n=1) involving PA apps only (n=5) used either mobile phone apps (3/5, 60%) or web-based applications (2/5, 40%) as their platform. The systematic reviews (n=5) and meta-analysis (n=7) involving PA+Diet apps used either mobile phone apps (8/12, 67%) or web-based applications (10/12, 83%) as their platform. Of note, the systematic reviews (n=9) and meta-analyses (n=8) involving PA and PA+Diet apps did not disclose the adherence rate of how frequently the participants used the interventions.

### CVD Risk Factors

The systematic reviews (n=9) involving PA and PA+Diet apps for the CVD risk factors outcomes are presented in Table 2. Of the systematic reviews (n=4) involving PA apps, the following CVD risk factors improved: body weight and BMI (2/4, 50%), BP (1/4, 25%), HbA_1c_ (1/4, 25%), and blood lipids (1/4, 25%) decreased, while PA (4/4, 100%) increased. Of the systematic reviews (n=5) involving PA+Diet apps, the following CVD risk factors improved: body weight and BMI (3/5, 60%), BP (1/5, 20%), and HbA_1c_ (3/5, 60%) decreased, while PA (3/5, 60%) increased.

**Table 2:** The influence of physical activity, and physical activity and diet apps on cardiovascular disease risk factors outcomes among the qualifying systematic reviews (n=9).

Outcome	PA^a^ apps		PA + Diet apps	
	Favors intervention, n	No difference intervention vs Control, n	Favors intervention, n	No difference in the intervention vs Control, n
BMI	1 [38]	1	1 [41]	2 [42,44]
Body weight	2 [36,38]	—^b^	2 [43,44]	—
Waist circumference	—	—	—	—
Systolic blood pressure	1 [38]	—	1 [44]	2 [41,42]
Diastolic blood pressure	—	—	1 [44]	2 [41,42]
Hemoglobin A_1c_	—	—	3 [40-42]	—
Fasting blood glucose	—	—	—	—
Total cholesterol	—	—	—	—
Triglycerides	—	—	—	1 [41]
High-density lipoprotein	1 [38]	—	—	—
Low-density lipoprotein	1 [38]	—	—	—
Physical activity measures	4 [36-39]	—	3 [40,41,44]	1 [42]

^a^PA: physical activity.

^b^Not available.

We categorized the effect sizes as (1) Favors PA or PA+Diet apps when the effect size was negative (or positive for HDL and PA) and statistically significant; (2) Favors Control when the effect size was positive (or negative for HDL and PA) and statistically significant; and (3) No difference between PA or PA+Diet apps vs Control when the effect size was not statistically significant. No systematic review was reported to favor control.

**Figure 2:**
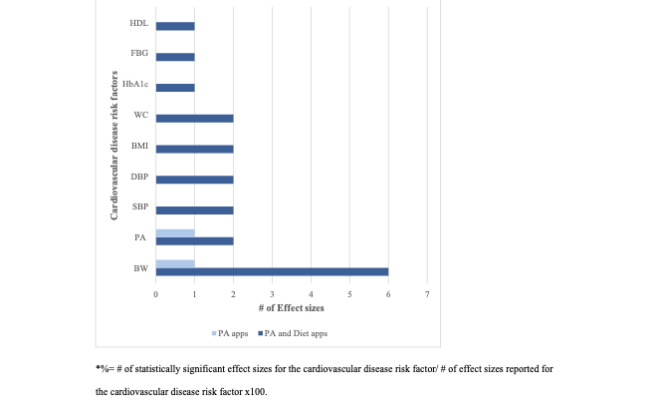
The number of statistically significant unstandardized and standardized effect sizes reported in the meta-analysis (n=8) for the improvement in cardiovascular disease risk factors by physical activity (PA) and physical activity and diet (PA+Diet) apps.

The meta-analyses (n=8) involving PA and PA+Diet apps unstandardized and standardized effect sizes for the CVD risk factors outcomes are presented in Table 3 and Figure 2. Of the meta-analyses reporting unstandardized effect sizes (n=1) involving PA apps, these CVD risk factors improved: body weight decreased (–0.73 kg, 95% CI –1.45 to –0.01; *P*=.05) and PA increased by 25 minutes/week (95% CI 0.58-1.68; *P*<.001) while BMI (–0.09 kg/m^2^, 95% CI –0.29 to 0.10; *P*=.35) and waist circumference (–1.92 cm, 95% CI –3.94 to 0.09; *P*=.06) tended to decrease. Of the meta-analyses reporting unstandardized effect sizes (n=4) involving PA+Diet apps, these CVD risk factors improved: body weight (4/4, 100%; effect sizes ranged from –1.79 kg (95% CI –3.17 to –0.41; *P*=.01) to –2.27 kg (95% CI –3.64 to –0.90; *P*<.01), BMI (1/4, 25%; effect size: –0.64 kg/m^2^, 95% CI –1.09 to –0.18, *P*=.01), waist circumference (1/4, 25%; effect size: –2.46 cm, 95% CI –4.56 to –0.36, *P*=.02), systolic/diastolic BP (1/4, 25%; effect size: –4.22/–2.87 mm Hg, 95% CI –6.54 to –1.91/ –4.44 to –1.29; *P*<.01), and HbA_1c_ (1/4, 25%; effect size: –0.43%, 95% CI –0.68 to –0.19, *P*<.001) decreased.

**Table 3:** The influence of physical activity and physical activity and diet apps on the cardiovascular disease risk factors among the qualifying meta-analyses (n=8).

Outcome	PA^a^ apps effect sizes		PA + Diet apps effect sizes	
	Favors intervention	No difference intervention vs Control^b^	Favors intervention	No difference intervention vs Control^b^
BMI	—^c^	n=1 [45]; unstandardized effect size: –0.09 kg/m^2^, 95% CI –0.29 to 0.10; *P*=.35	n=2 [46,48]; standardized effect size: –0.46, 95% CI –0.68 to –0.23; *P*=.04; unstandardized effect size: –0.64 kg/m^2^, 95% CI –1.09 to –0.18; *P*=.01	n=1 [52]; standardized effect size: –0.14 95% CI –0.51 to 0.23; *P*=.45
Body weight	n=1 [45]; unstandardized effect size: –0.73 kg, 95% CI –1.45-0.01; *P*=.05	—	n=6 [47-52]; standardized effect size n=2: –0.37, 95% CI –0.54 to –0.19; *P*<.001 to –0.43, 95% CI –0.252 to –0.609; *P*<.01; unstandardized effect size n=4: –1.79 kg 95% CI –3.17 to 0.41; *P*=.01 to –2.27 kg 95% CI –3.64 to –0.90; *P*<.01	—
Waist circumference	—	n=1 [45]; unstandardized effect size: –1.92 cm, 95% CI –3.94 to 0.09; *P*=.06	n=2 [46,48]; standardized effect size: –0.54, 95% CI –0.85 to –0.23; *P*=.001; unstandardized effect size: –2.46 cm 95% CI –4.56 to –0.36; *P*=.02	—
Systolic blood pressure	—	—	n=2 [46,48]; standardized effect size: –0.43, 95% CI –0.77 to –0.10; *P*<.001; unstandardized effect size: –4.22 mm Hg 95% CI –6.54 to –1.91; *P*<.01	—
Diastolic blood pressure	—	—	n=2 [46,48]; standardized effect size: –0.44, 95% CI –0.74 to –0.14; *P*=.002; unstandardized effect size: –2.87 mm Hg 95% CI –4.44 to –1.29; *P*<.01	—
Hemoglobin A_1c_	—	—	n=1 [49]; unstandardized effect size: –0.43% 95% CI –0.6 to –0.19; *P*<.001	n=1 [46]; standardized effect size: –0.35, 95% CI –0.82 to 0.12, *P*<.001
Fasting blood glucose	—	—	n=1 [46]; standardized effect size: –0.39, 95% CI –0.73 to –0.06; *P*=.002	—
Total cholesterol	—	—	—	n=1 [46]; standardized effect size: –0.06, 95% CI –0.23 to 0.11; *P*=.47
Triglycerides	—	—	—	n=1 [46]; standardized effect size: –0.20, 95% CI –0.35 to –0.04; *P*=.80
High-density lipoprotein	—	—	n=1 [46]; standardized effect size: 0.23, 95% CI 0.07-0.38; *P*=.50	—
Low-density lipoprotein	—	—	—	n=1 [46]; standardized effect size: –0.04, 95% CI –0.21 to 0.13; *P*=.96
Physical activity measures	n=1 [45]; standardized effect size: 1.13 (~25 minutes/week moderate to vigorous PA), 95% CI 0.58-1.68; *P*<.001	—	n=2 [49,52]; standardized effect size: 0.21, 95% CI 0.05-0.37; *P*=.01 to 2.59, 95% CI 1.00-4.81; *P*=.001	—

^a^PA: physical activity.

^b^No effect size was reported to favor control.

^c^Not available.

Of the meta-analyses reporting standardized effect sizes (n=3) involving PA+Diet apps, these CVD risk factors improved: body weight (2/3, 66%; effect sizes ranged from –0.37 (95% CI –0.54 to –0.19; *P*<.001) to –0.43 (95% CI –0.252 to –0.609; *P*<.01), BMI (1/3, 33%; effect size: –0.46, 95% CI –0.68 to –0.23; *P*=.04), waist circumference (1/3, 33%; effect size: –0.54, 95% CI –0.85 to –0.23; *P*=.001), systolic/diastolic BP (1/3, 33%; effect size: –0.43/–0.44, 95% CI –0.77 to –0.10/ –0.74 to –0.14; *P*<.001; *P*=.002), fasting blood glucose (1/3, 33%; effect size: –0.39, 95% CI –0.73 to –0.06; *P*=.002), HDL (1/3, 33%; effect size: 0.23, 95% CI 0.07-0.38, *P*=.50), and PA (2/3, 66%; effect sizes ranged from 0.21 (95% CI 0.05-0.37, *P*=.01) to 2.59 (95% CI 1.00-4.81, *P*=.001); while HbA_1c_ (1/3, 33%; effect size: –0.35, 95% CI –0.82 to 0.12; *P*<.001), total cholesterol (1/3, 33%; effect size: –0.06, 95% CI –0.23 to 0.11; *P*=.47), triglycerides (1/3, 33%; effect size: –0.20, 95% CI –0.35 to –0.04; *P*=.80), and LDL (1/3, 33%; effect size: –0.04, 95% CI –0.21 to 0.13; *P*=.96) tended to decrease.

### Study Methodological Quality Assessment

The overall methodological study quality of the literature was moderate to high, with 35% scoring low, 29% moderate, and 35% high on the AMSTAR Checklist. Given the overall study, the methodological study quality was moderate to high; we conclude there is moderate certainty of evidence in our findings. See Multimedia Appendix 2 for a summary of the methodological quality assessment from the AMSTAR Checklist.

## Discussion

### Principal Findings

Our meta-review provides the first evidence synthesis of systematic reviews and meta-analyses examining the influence of PA and PA+Diet apps on the major CVD risk factors of physical inactivity, obesity, DM, dyslipidemia, and hypertension. For the systematic reviews involving PA apps, body weight and BMI improved compared to control. For the meta-analyses involving PA apps, body weight decreased by 0.73 kg compared with control. For the systematic reviews involving PA apps, HbA_1c_ improved compared with control. For the systematic reviews involving PA apps, blood lipids improved compared to control. For the systematic reviews involving PA apps, PA improved compared to control. For the meta-analyses involving PA apps, PA increased by 25 minutes/week compared to control. For the systematic reviews involving PA+Diet apps, body weight and BMI improved compared to control. For the meta-analyses involving PA+Diet apps, BMI decreased by 0.64 kg/m^2^, and waist circumference decreased by 2.46 cm compared to control. For the systematic reviews involving PA+Diet apps, BP improved compared to control. For the meta-analyses involving PA+Diet apps, BP decreased by 3-4 mm Hg compared to control. For the systematic reviews involving PA+Diet apps, HbA_1c_ improved compared to control. For the meta-analyses involving PA+Diet Apps, HbA_1c_ decreased by 0.40 units compared to control. For the systematic reviews involving PA+Diet apps, PA improved compared to control. We found that 70% of the included systematic reviews and meta-analyses had moderate to high study methodological quality ratings on the AMSTAR Checklist [34]. Our meta-review suggests that both PA and PA+Diet apps resulted in clinically meaningful improvements in major CVD risk factor outcomes. However, the size of the literature involving systematic reviews and meta-analyses of PA+Diet apps (n=12) is larger than PA apps (n=5), and the findings were more robust for anthropometric and PA outcomes than the other major CVD risk factor outcomes of BP, fasting blood glucose, HbA_1c_, and HDL.

### Comparison With Previous Works

Consistent with our findings, other studies have shown that even modest reductions in anthropometric CVD risk factor outcomes (ie, body weight, BMI, and waist circumference) can lead to clinically important improvements in other CVD risk factor outcomes [17,53-55]. Aucott et al [56] found a 4.5-kg decrease in body weight was associated with improvements in multiple CVD risk factors, including BP, blood lipids, and fasting blood glucose levels. Aune et al [15] and Zhou [57] found a 1-kg/m^2^ decrease in BMI and a 1-cm decrease in waist circumference [57] associated with significant improvements in BP, total cholesterol, fasting blood glucose, and triglyceride levels [15] and a 2% to 4% decrease in the risk of developing CVD [57]. The decrease in body weight of 2.27 kg, BMI of 0.64 kg/m^2^, and waist circumference of 2.46 cm resulting from PA+Diet apps reported by McMahon et al [48] in our meta-review suggest the improvements in measures of body composition they observed have the potential to positively impact other CVD risk factors. Lear et al [54] found that participating in 30 minutes of daily PA was associated with a 27% lower risk of CVD incidence and a 30% lower risk of CVD mortality than being physically inactive. The finding of increases in PA of 25 minutes/week resulting from PA apps reported by Cotie et al [45] in our meta-review is not as large as Lear et al [54] but this amount of weekly PA would have positive effects on the risk of CVD incidence and mortality as well [45]. Lewington et al [58] found a 3-mm Hg reduction in SBP and a 2-mm Hg reduction in DBP led to a 5%-6% reduction in the risk of CVD events. The finding of decreases in BP of 3-4 mm Hg resulting from PA+Diet apps reported by McMahon et al. [48] in our meta-review suggests they, too, may reduce the risk of CVD events. Improving one or multiple CVD risk factors can significantly reduce the risk of developing CVD [9-11]. A meta-analysis performed on the influence of body weight and BP found that body weight reductions of 5.1 kg through diet, PA, or both reduced SBP by –4.44 mm Hg and DBP by –3.57 mm Hg [10]. In addition, increases in PA tend to increase HDL by 1-2 mg/dl and decrease triglycerides by –4 to 12 mg/dl [11]. Collectively, our meta-review findings provide promising evidence that PA and PA+Diet apps that include behavioral change strategy features may lead to clinically important improvements in major CVD risk factor outcomes and overall cardiovascular health.

The PA and PA+Diet apps systematic reviews (n=9) and meta-analyses (n=8) in our meta-review integrated a variety of behavioral change strategies, the most common of which were personalized feedback (8/17, 47%), self-monitoring (7/17, 41%), and goal setting (5/17, 29%). The systematic reviews (n=4) and meta-analyses (n=1) involving PA apps primarily used personalized feedback (1/4, 20%), goal setting (1/4, 20%), and PA reminders (1/4, 20%), while the systematic reviews (n=5) and meta-analyses (n=7) involving PA+Diet apps primarily used personalized feedback (7/12, 58%), self-monitoring (7/12, 58%), and goal setting (4/12, 33%). All major professional organizations recommend the integration of behavioral change strategies in interventions to promote healthy lifestyles [59]. Michie et al [60] developed the Behavior v1 Change Technique Taxonomy, a classification system based upon an expert consensus of the reliable and transparent behavior change strategies that can be used by practitioners with confidence. Out of the 93 distinct behavioral change strategies included in their classification system, those with some of the highest levels of interrater agreement were self-monitoring, goal setting, and feedback. The systematic reviews and meta-analyses in our meta-review, consistent with their overall moderate to high study methodological quality, integrated some of the most highly regarded behavioral change strategies from this classification scheme within their PA and PA+Diet app interventions. However, the most common intervention duration was relatively short (6 and 8 weeks). Given the shorter duration, it remains unclear to what extent the behavioral change strategies were an “active ingredient” in the CVD risk factor improvements that resulted from the PA and PA+Diet app interventions [60]. It is also unclear from the systematic reviews and meta-analyses in out meta-review if there are long-term effects that are sustained following these PA and PA+Diet app interventions [60].

CVD remains the leading cause of death in the United States, accounting for 928,741 deaths in the year 2020 [1]. The PA and PA+Diet app market to prevent and treat CVD and its risk factors has escalated because they are cost-effective, easy to use, accessible, and time-efficient [12-14]. The mobile health app market was valued at US $25.92 billion in 2020 and is expected to grow to US $348.98 billion by 2027 [12]. PA+Diet apps have dominated the mobile health app market with a share of 54.6% in 2022 [13]. The favorable findings from this meta-review on the use of PA and PA+Diet apps to improve major CVD risk factors combined with the growth of the mobile health app market indicates the future potential of this industry to favorably impact CVD and its risk factors is untapped [12-14].

### Strengths and Limitations

There are limitations to this meta-review. First, baseline values of the CVD risk factor outcomes were not disclosed. It is well established that the response of a CVD risk factor is a direct function of baseline values [61], except perhaps HDL [62]. Baseline values are necessary for the interpretation of the response to health outcomes as the response of a health outcome is a direct function of the baseline value, which is also needed to determine the relative percentage of improvement over the course of the app interventions. Since baseline values were not provided, it is difficult to fully appreciate the clinical implications of the resultant CVD risk factor changes. Second, the systematic reviews and meta-analyses did not disclose ethnicity, nor did they report the frequency of app use within the intervention period. Third, non-English studies were omitted from this review. Despite these limitations, our meta-review has several strengths. The systematic search methods adhered to the contemporary PRISMA standards [28,29] and included the assessment of the study's methodological quality [34]. Finally, to the best of our knowledge, this meta-review provides the first evidence synthesis of systematic reviews and meta-analyses examining the influence of PA and PA+Diet apps on the major CVD risk factors of physical inactivity, obesity, DM, dyslipidemia, and hypertension.

### Future Directions

In conclusion, we found PA and PA+Diet apps lead to improvements in anthropometric and PA outcomes with favorable but less consistent effects on other CVD risk factors outcomes of BP, fasting blood glucose, HbA_1c_, and HDL. These findings hold promise because the prevalence of CVD and its risk factors are so high [1,2,4-8], and the health apps market is proliferating [13]. Despite the positive findings of our meta-review, there is a need for future RCTs that directly compare and quantify the effects of PA and PA+Diet apps on CVD risk factor outcomes and evaluate which behavioral strategies within them are most effective. In this way, the prevention and treatment of CVD can capitalize on the momentum of the rapidly growing health app market.

## Appendix

Multimedia Appendix 1 A Summary of the Secondary Literature Systematic Search Strategy.

Multimedia Appendix 2 A Summary of the Assessment of the Study Methodological Quality of the Included Meta-Analyses (N=17).

Multimedia Appendix 3 PRISMA (Preferred Reporting Items for Systematic Reviews and Meta-Analyses) checklist.

Multimedia Appendix 4 A Summary of the Sample, Study, and Intervention Characteristics of the included RCTs within the Qualifying Systematic Reviews and Meta-Analyses (N=17).

## Abbreviations

AMSTAR: A Measurement Tool for Assessment of Multiple Systematic Reviews
BCT: behavioral change technique
BP: blood pressure
CVD: cardiovascular disease
DBP: diastolic blood pressure
DM: diabetes mellitus
HbA1c: hemoglobin A1c
HDL: high-density lipoprotein
LDL: low-density lipoprotein
PA: physical activity
PA+Diet: physical activity and diet
PRISMA: Preferred Reporting Items for Systematic Reviews and Meta-Analyses
RCT: randomized controlled trial
SBP: systolic blood pressure
