# Supplementary material for: The Influence of Physical Activity and Diet Mobile Apps on Cardiovascular Disease Risk Factors: Meta-Review
Source: J Med Internet Res. 2024 Oct 9;26:e51321. doi: 10.2196/51321 (PMC11499721; doi:10.2196/51321)
Supplement: Multimedia Appendix 1 [file jmir_v26i1e51321_app1.docx]

**Multimedia Appendix 1. A Summary of the Secondary Literature Systematic Search Strategy**

Original PubMed searches run from inception to January 12, 2022. Total Citations: 131

Original Cochrane searches run from inception to January 12, 2022. Total Citations: 111

Original CINAHL searches run from inception to January 12, 2022. Total Citations: 178

Original SportDiscus searches run from inception to January 12, 2022. Total Citations: 14

Original Scopus searches run from inception to January 12, 2022. Total Citations: 373

**PubMed Search**

**Mobile application AND Exercise AND systematic reviews/meta-analysis NOT infant NOT animal**

**Inception to January 12, 2022. Citations: 131**

Mobile application* [tiab] OR Mobile apps [tiab] OR Mobile health application* [tiab] OR Mobile health apps [tiab] OR Mobile tech* [tiab] OR Mobile health tech* [tiab] OR Cell phone application* [tiab] OR Cell phone apps [tiab] OR Smartphone application* [tiab] OR Smartphone apps [tiab] OR iPhone application* [tiab] OR iPhone apps [tiab] OR Android application* [tiab] OR Android apps [tiab] OR mHealth [tiab] OR Mobile App [tiab] OR Portable Electronic App* [tiab] OR Portable Electronic Application* [tiab] OR Portable Software Apps [tiab] OR Portable Software App [tiab] OR Portable Software Application* [tiab] OR Mobile health [tiab] OR eHealth [tiab] OR Exercise mobile application* [tiab] OR Consumer exercise application* [tiab] OR Consumer fitness application* [tiab] Google Play [tiab] OR App store [tiab] OR Internet [tiab] OR Telephone* [tiab] OR Mobile applications [mesh] OR Cell phone [mesh] OR Smartphone [mesh] OR Internet [mesh] OR Telephone [mesh]

AND

Exercise* [tiab] OR Physical Activity [tiab] OR Activities, Physical [tiab] OR Activity, Physical [tiab] OR Physical Activities [tiab] OR Exercise, Physical [tiab] OR Exercises, Physical [tiab] OR Physical Exercise [tiab] OR Physical Exercises [tiab] OR Acute Exercise* [tiab] OR Exercise, Acute [tiab] OR Exercises, Acute [tiab] OR Exercise, Isometric [tiab] OR Exercises, Isometric [tiab] OR Isometric Exercises [tiab] OR Isometric Exercise [tiab] OR Exercise, Aerobic [tiab] OR Aerobic Exercise* [tiab] OR Exercises, Aerobic [tiab] OR Exercise Training* [tiab] OR Training, Exercise [tiab] OR Trainings, Exercise [tiab] OR Exercise [mesh] OR Physical Activity [mesh] OR Physical Activities [mesh]

AND

Blood pressure [tiab] OR High blood pressure* [tiab] OR Systolic blood pressure [tiab] OR Systolic pressure [tiab] OR Diastolic blood pressure [tiab] OR Diastolic pressure [tiab] OR Hypertension [tiab] OR Hypertensive [tiab] OR Antihypertensive [tiab] OR Blood glucose [tiab] OR Diabetes [tiab] OR High blood glucose [tiab] OR Waist circumference* [tiab] OR BMI [tiab] OR Body mass index [tiab] OR Weight [tiab] OR Diabetes Mellitus [tiab] OR Insulin Resistance [tiab] OR Dyslipidemia [tiab] OR Elevated blood pressure [tiab] OR Pre-Hypertension [tiab] OR Pre Hypertension [tiab] OR Venous Blood Pressure* [tiab] OR Blood sugar [tiab] OR Glucose Metabolism Disorder [tiab] OR Glucose Metabolic Disorder [tiab] OR Hyperglycemias [tiab] OR Body Weights [tiab] OR Quetelet Index [tiab] OR Quetelet's Index [tiab] OR Quetelets Index [tiab] OR Insulin Sensitivity [tiab] OR Dyslipidemia [tiab] OR Dyslipoproteinemia* [tiab] OR Lipid* [tiab] OR High density lipoprotein [tiab] OR Low density lipoprotein [tiab] OR Total cholesterol [tiab] OR Cholesterol [tiab] OR HDL Lipoproteins [tiab] OR High-Density Lipoprotein* [tiab] OR High Density Lipoprotein* [tiab] OR alpha-Lipoprotein* [tiab] OR alpha Lipoprotein* [tiab] OR Heavy Lipoproteins [tiab] OR alpha-1 Lipoprotein [tiab] OR LDL Lipoproteins [tiab] OR beta-Lipoprotein [tiab] OR beta Lipoprotein [tiab] OR Low-Density Lipoprotein* [tiab] OR Low Density Lipoprotein* [tiab] OR beta-Lipoproteins [tiab] OR beta Lipoproteins [tiab] OR LDL-2 [tiab] OR LDL2 [tiab] OR Low-Density Lipoprotein 2 [tiab] OR Low Density Lipoprotein 2 [tiab] OR LDL(2) OR LDL-1 [tiab] OR LDL1 [tiab] OR Low-Density Lipoprotein 1 [tiab] OR Low Density Lipoprotein 1 [tiab] OR LDL(1) OR Blood lipid [tiab] OR Triacylglycerol [tiab] OR Triacylglycerols [tiab] OR Triglyceride [tiab] OR Total Cholesterol [tiab] OR Blood pressure [mesh] OR Hypertension [mesh] OR Arterial pressure [mesh] OR Hypotension [mesh] OR Prehypertension [mesh] OR Venous pressure [mesh] OR Blood glucose [mesh] OR Diabetes Mellitus [mesh] OR Glucose Metabolism Disorders [mesh] OR Hyperglycemia [mesh] OR Myocardial Infarction [mesh] OR Waist circumference [mesh] OR Body weight [mesh] OR Body mass index [mesh] OR Atherosclerosis [mesh] OR Insulin resistance [mesh] OR Dyslipidemias [mesh] OR Lipids [mesh] OR Lipoproteins [mesh] OR Lipoproteins, HDL [mesh] OR Lipoproteins, LDL [mesh] OR Cholesterol [mesh] OR Triglycerides [mesh] OR Heart Disease [mesh]

AND

"systematic"[filter] OR "meta-analysis"[pt] OR "meta-analysis as topic"[mh] OR meta analy*[tw] OR metanaly*[tw] OR metaanaly*[tw] OR met analy*[tw] OR integrative research[tiab]  OR integrative review*[tiab] OR integrative overview*[tiab] OR research integration*[tiab] OR research overview*[tiab] OR collaborative review*[tiab] OR collaborative overview*[tiab] OR "systematic review"[pt] OR "systematic reviews as topic"[mh] OR systematic review*[tiab] OR technology assessment*[tiab] OR technology overview*[tiab] OR technology appraisal*[tiab] OR "Technology Assessment, Biomedical"[mh] OR HTA[tiab] OR HTAs[tiab] OR comparative efficacy[tiab] OR comparative effectiveness[tiab] OR outcomes research[tiab] OR indirect comparison*[tiab] OR Bayesian comparison[tiab] OR ((indirect treatment[tiab] OR mixed-treatment[tiab]) AND comparison*[tiab]) OR Embase*[tiab] OR Cinahl*[tiab] OR systematic overview*[tiab] OR methodological overview*[tiab]  OR methodologic overview*[tiab]  OR methodological review*[tiab]  OR methodologic review*[tiab] OR quantitative review*[tiab] OR  quantitative overview*[tiab] OR quantitative synthes*[tiab] OR pooled analy*[tiab] OR Cochrane[tiab] OR Medline[tiab] OR Pubmed[tiab] OR Medlars[tiab] OR handsearch*[tiab] OR hand search*[tiab] OR meta-regression*[tiab] OR metaregression*[tiab] OR data synthes*[tiab] OR data extraction[tiab] OR data abstraction*[tiab] OR mantel haenszel[tiab] OR peto[tiab] OR der-simonian[tiab] OR dersimonian[tiab] OR fixed effect*[tiab] OR multiple treatment comparison[tiab] OR mixed treatment meta-analys*[tiab] OR umbrella review*[tiab] OR ((multiple paramet*[tiab]) AND (evidence synthesis[tiab]))  OR ((multi-paramet*[tiab]) AND (evidence synthesis[tiab])) OR ((multiparameter*[tiab]) AND (evidence synthesis[tiab])) OR "Cochrane Database Syst Rev"[Journal] OR "health technology assessment winchester, england"[Journal] OR  "Evid Rep Technol Assess (Full Rep)"[Journal] OR "Evid Rep Technol Assess (Summ)"[Journal] OR "Int J Technol Assess Health Care"[Journal] OR "GMS Health Technol Assess"[Journal] OR "Health Technol Assess (Rockv)"[Journal] OR "Health Technol Assess Rep"[Journal]

NOT

Infan* [tiab] OR newborn* [tiab] OR new-born* [tiab] OR perinat* [tiab] OR neonat* [tiab] OR baby [tiab] OR baby* [tiab] OR babies [tiab] OR toddler* [tiab] OR kid [tiab] OR kids [tiab] OR child [tiab] OR child* [tiab] OR children* [tiab] OR schoolchild* [tiab] OR schoolchild [tiab] OR school child [tiab] OR school child* [tiab] OR youth* [tiab] OR teen* [tiab] OR pubescen* [tiab] OR pediatrics [tiab] OR pediatric* [tiab] OR paediatric* [tiab] OR peadiatric* [tiab] OR school [tiab] OR school* [tiab] OR prematur* [tiab] OR preterm* [tiab]

NOT

("animals"[MeSH Terms] NOT "humans"[MeSH Terms])

**Cochrane Search**

**Mobile application AND Exercise**

**Inception to January 12, 2022. Citations: 111**

(Mobile NEXT app*) OR (Mobile NEXT health NEXT app*) OR (Mobile NEXT tech*) OR (Mobile NEXT health NEXT tech*) OR (Cell* NEXT phone NEXT app*) OR (Smartphone NEXT app*) OR (iPhone NEXT app*) OR (Android NEXT app*) OR mHealth OR (Portable NEXT Electronic NEXT App*) OR (Portable NEXT Software NEXT App*) OR (Mobile NEXT health) OR eHealth OR (Clinical NEXT Decision NEXT Support*) OR (Exercise NEXT mobile NEXT application*) OR (Consumer NEXT exercise NEXT application*) OR (Consumer NEXT fitness NEXT application*) OR (Google NEXT play) OR (App NEXT Store) OR (Internet) OR (Telephone)

OR

[mh “Mobile Applications”] OR [mh “cell phone”] OR [mh smartphone] OR[mh Internet] OR [mh Telephone]

AND

(Exercise*) OR (Physical NEXT Activity) OR (Physical NEXT Activities) OR (Physical NEXT Exercise*) OR (Acute NEXT Exercise*) OR (Isometric NEXT Exercise*) OR (Aerobic NEXT Exercise*) OR (Exercise NEXT Training*)

OR

[mh “Exercise”]

AND

((Blood NEXT pressure) OR (High NEXT blood NEXT pressure*) OR (Systolic NEXT blood NEXT pressure) OR (Systolic NEXT pressure) OR (Diastolic NEXT blood NEXT pressure) OR (Diastolic NEXT pressure) OR Hypertension OR Hypertensive OR Antihypertensive OR Prehypertension* OR Prehypertensive OR (Blood NEXT glucose) OR Diabetes OR (High NEXT blood NEXT glucose) OR (Waist NEXT circumference*) OR BMI OR (Body NEXT mass NEXT index) OR Weight OR (Diabetes NEXT Mellitus) OR (Insulin NEXT Resistance) OR Dyslipidemia OR (Elevated NEXT blood NEXT pressure) OR (Insulin NEXT Resistance NEXT Syndrome NEXT X) OR Pre-Hypertension OR (Pre NEXT Hypertension) OR (Venous NEXT Blood NEXT Pressure*) OR (Blood NEXT sugar) OR (Glucose NEXT Metabolism NEXT Disorder) OR (Glucose NEXT Metabolic NEXT Disorder) OR (Body NEXT Weights) OR (Quetelet NEXT Index) OR (Quetelet's NEXT Index) OR (Quetelets NEXT Index) OR (Insulin NEXT Sensitivity) OR Dyslipidemia OR Dyslipoproteinemia* OR Lipid* OR (High NEXT density NEXT lipoprotein) OR (Low NEXT density NEXT lipoprotein) OR (Total NEXT cholesterol) OR Cholesterol OR (HDL NEXT Lipoproteins) OR (High-Density NEXT Lipoprotein*) OR (High NEXT Density NEXT Lipoprotein*) OR alpha-Lipoprotein* OR (alpha NEXT Lipoprotein*) OR (Heavy NEXT Lipoproteins) OR (alpha-1 NEXT Lipoprotein) OR (LDL NEXT Lipoproteins) OR beta-Lipoprotein OR (beta NEXT Lipoprotein) OR (Low-Density NEXT Lipoprotein*) OR (Low NEXT Density NEXT Lipoprotein*) OR beta-Lipoproteins OR (beta NEXT Lipoproteins) OR LDL-2 OR LDL2 OR (Low-Density NEXT Lipoprotein NEXT 2) OR (Low NEXT Density NEXT Lipoprotein NEXT 2) OR LDL(2) OR LDL-1 OR LDL1 OR (Low-Density NEXT Lipoprotein NEXT 1) OR (Low NEXT Density NEXT Lipoprotein NEXT 1) OR LDL(1) OR (Blood NEXT lipid) OR Triacylglycerol OR Triacylglycerols OR Triglyceride OR (Total NEXT Cholesterol))

OR

[mh “Blood Pressure”] OR [mh “Hypertension”] OR [mh “Arterial Pressure”] OR [mh Prehypertension] OR [mh “Diabetes Mellitus”] OR [mh “Glucose Metabolism Disorders”] OR [mh “Waist Circumference”] OR [mh “Body Weight”] OR [mh “Body Mass Index”] OR [mh “Insulin Resistance”] OR [mh Dyslipidemias] OR [mh Lipids] OR [mh Lipoproteins] OR [mh “Lipoproteins, HDL”] OR [mh “Lipoproteins, LDL”] OR [mh Cholesterol] OR [mh Triglycerides]

**SportDiscus and CIHAHL Search**

**Mobile application AND Exercise AND systematic reviews/meta-analysis**

**Inception to January 12, 2022. Citations: 178 (CINAHL), 14 (SportDiscus)**

“Mobile application*” OR “Mobile apps” OR “Mobile health application*” OR “Mobile health apps” OR “Mobile tech*” OR “Mobile health tech*” OR “Cell phone application*” OR “Cell phone apps” OR “Smartphone application*” OR “Smartphone apps” OR “iPhone application*” OR “iPhone apps” OR “Android application*” OR “Android apps” OR “mHealth” OR “Mobile App” OR “Portable Electronic App*” OR “Portable Electronic Application*” OR “Portable Software Apps” OR “Portable Software App” OR “Portable Software Application*” OR “Mobile health” OR “eHealth” OR “Exercise mobile application*” OR “Consumer exercise application*” OR “Consumer fitness application*” OR “Google Play” OR “App store” OR “Mobile applications” OR “Cell phone” OR “Smartphone” OR “Internet” OR “Telephone”

AND

“Exercise*” OR “Physical Activity” OR “Activities, Physical” OR “Activity, Physical” OR “Physical Activities” OR “Exercise, Physical” OR “Exercises, Physical” OR “Physical Exercise” OR “Physical Exercises” OR “Acute Exercise*” OR “Exercise, Acute” OR “Exercises, Acute” OR “Exercise, Isometric” OR “Exercises, Isometric” OR “Isometric Exercises” OR “Isometric Exercise” OR “Exercise, Aerobic” OR “Aerobic Exercise*” OR “Exercises, Aerobic” OR “Exercise Training*” OR “Training, Exercise” OR “Trainings, Exercise”

AND

“Blood pressure” OR “High blood pressure*” OR “Systolic blood pressure” OR “Systolic pressure” OR “Diastolic blood pressure” OR “Diastolic pressure” OR “Hypertension” OR “Hypertensive” OR “Antihypertensive” OR “Blood glucose” OR “Diabetes” OR “High blood glucose” OR “Waist circumference*” OR “BMI” OR “Body mass index” OR “Weight” OR “Diabetes Mellitus” OR “Insulin Resistance” OR “Dyslipidemia” OR “Elevated blood pressure” OR “Pre-Hypertension” OR “Pre Hypertension” OR “Venous Blood Pressure*” OR “Blood sugar” OR “Glucose Metabolism Disorder” OR “Glucose Metabolic Disorder” OR “Hyperglycemias” OR “Body Weights” OR “Quetelet Index” OR “Quetelet's Index” OR “Quetelets Index” OR “Insulin Sensitivity” OR “Dyslipidemia” OR “Dyslipoproteinemia*” OR “Lipid*” OR “High density lipoprotein” OR “Low density lipoprotein” OR “Total cholesterol” OR “Cholesterol” OR “HDL Lipoproteins” OR “High-Density Lipoprotein*” OR “High Density Lipoprotein*” OR “alpha-Lipoprotein*” OR “alpha Lipoprotein*” OR “Heavy Lipoproteins” OR “alpha-1 Lipoprotein” OR “LDL Lipoproteins” OR “beta-Lipoprotein” OR “beta Lipoprotein” OR “Low-Density Lipoprotein*” OR “Low Density Lipoprotein*” OR “beta-Lipoproteins” OR “beta Lipoproteins” OR “LDL-2” OR “LDL2” OR “Low-Density Lipoprotein 2” OR “Low Density Lipoprotein 2” OR “LDL(2)” OR “LDL-1” OR “LDL1” OR “Low-Density Lipoprotein 1” OR “Low Density Lipoprotein 1” OR “LDL(1)” OR “Blood lipid” OR “Triacylglycerol” OR “Triacylglycerols” OR “Triglyceride” OR “Total Cholesterol” OR “Arterial pressure” OR “Hypotension” OR “Prehypertension” OR “Venous pressure” OR “Blood glucose” OR “Glucose Metabolism Disorders” OR “Hyperglycemia” OR “Myocardial Infarction” OR “Body weight” OR “Atherosclerosis” OR “Dyslipidemias” OR “Lipids” OR “Lipoproteins” OR “Lipoproteins, HDL” OR “Lipoproteins, LDL” OR “Heart Disease”

AND (CINAHL Only)

(MH meta analysis OR MH systematic review OR MH "Technology, Medical/EV" OR PT systematic review OR PT meta analysis OR systematic* N3 review* OR systematic* N3 overview* OR methodologic* N3 review* OR methodologic* N3 overview* OR quantitative N3 review* OR quantitative N3 overview* OR quantitative N3 synthes* OR research N3 integrati* OR research N3 overview* OR integrative N3 review* OR integrative N3 overview* OR collaborative N3 review* OR collaborative N3 overview* OR pool* N3 analy* OR TI data synthes* OR AB data synthes* OR TI data extraction* OR AB data extraction* OR TI data abstraction* OR AB data abstraction* OR TI handsearch* OR AB handsearch* OR TI hand search* OR AB hand search* OR TI mantel haenszel OR AB mantel haenszel OR TI peto OR AB peto OR TI der simonian OR AB der simonian OR TI dersimonian OR AB dersimonian OR TI fixed effect* OR AB fixed effect* OR TI latin square* OR AB latin square* OR TI meta analy* OR AB meta analy* OR MW meta analy* OR TI metaanaly* OR AB metaanaly* OR MW metaanaly* OR TI health technology assessment* OR AB health technology assessment* OR TI hta OR AB hta OR TI htas OR AB htas OR TI meta regression* OR AB meta regression* OR TI metaregression* OR AB metaregression* OR TI mega regression* OR AB mega regression* OR MW systematic review* OR MW biomedical technology assessment* OR MW bio-medical technology assessment* OR TI medline OR AB medline OR TI Cochrane OR AB Cochrane OR TI pubmed OR AB pubmed OR TI medlars OR AB medlars OR MW medline OR MW Cochrane OR MW pubmed OR MW medlars OR SO Cochrane OR SO health technology assessment OR SO evidence report or CF Y)

**Scopus Search**

**Mobile application AND Exercise AND systematic reviews/meta-analysis**

**Inception to January 12, 2022. Citations: 373**

( "Mobile application*"  OR  "Mobile apps"  OR  "Mobile health application*"  OR  "Mobile health apps"  OR  "Mobile tech*"  OR  "Mobile health tech*"  OR  "Cell phone application*"  OR  "Cell phone apps"  OR  "Smartphone application*"  OR  "Smartphone apps"  OR  "iPhone application*"  OR  "iPhone apps"  OR  "Android application*"  OR  "Android apps"  OR  mhealth  OR  "Mobile App"  OR  "Portable Electronic App*"  OR  "Portable Electronic Application*"  OR  "Portable Software Apps"  OR  "Portable Software App"  OR  "Portable Software Application*"  OR  "Mobile health"  OR  ehealth  OR  "Exercise mobile application*"  OR  "Consumer exercise application*"  OR  "Consumer fitness application*"  OR  "Google Play"  OR  "App store"  OR  "Mobile applications"  OR  "Cell phone"  OR  Smartphone OR Internet OR Telephone )

AND

(exercise* OR “Physical Activity” OR “Physical Activities” OR “Physical Exercise” OR “Physical Exercises” OR “Acute Exercise*" OR "Isometric Exercises" OR "Isometric Exercise" OR "Aerobic Exercise*" OR "Exercise Training*")

AND

( "Blood pressure"  OR  "High blood pressure*"  OR  "Systolic blood pressure"  OR  "Systolic pressure"  OR  "Diastolic blood pressure"  OR  "Diastolic pressure"  OR  hypertension  OR  hypertensive  OR  antihypertensive  OR  "Blood glucose"  OR  diabetes  OR  "High blood glucose"  OR  "Waist circumference*"  OR  bmi  OR  "Body mass index"  OR  weight  OR  "Diabetes Mellitus"  OR  "Insulin Resistance"  OR  dyslipidemia  OR  "Elevated blood pressure"  OR  "Pre-Hypertension"  OR  "Pre Hypertension"  OR  "Venous Blood Pressure*"  OR  "Blood sugar"  OR  "Glucose Metabolism Disorder"  OR  "Glucose Metabolic Disorder"  OR  hyperglycemias  OR  "Body Weights"  OR  "Quetelet Index"  OR  "Quetelet's Index"  OR  "Quetelets Index"  OR  "Insulin Sensitivity"  OR  dyslipoproteinemia*  OR  lipid*  OR  "High density lipoprotein"  OR  "Low density lipoprotein"  OR  "Total cholesterol"  OR  cholesterol  OR  "HDL Lipoproteins"  OR  "High-Density Lipoprotein*"  OR  "High Density Lipoprotein*"  OR  "alpha-Lipoprotein*"  OR  "alpha Lipoprotein*"  OR  "Heavy Lipoproteins"  OR  "alpha-1 Lipoprotein"  OR  "LDL Lipoproteins"  OR  "beta-Lipoprotein"  OR  "beta Lipoprotein"  OR  "Low-Density Lipoprotein*"  OR  "Low Density Lipoprotein*"  OR  "beta-Lipoproteins"  OR  "beta Lipoproteins"  OR  ldl-2  OR  ldl2  OR  "Low-Density Lipoprotein 2"  OR  "Low Density Lipoprotein 2"  OR  ldl-1  OR  ldl1  OR  "Low-Density Lipoprotein 1"  OR  "Low Density Lipoprotein 1"  OR  "Blood lipid"  OR  triacylglycerol  OR  triacylglycerols  OR  triglyceride  OR  "Arterial pressure"  OR  hypotension  OR  prehypertension  OR  "Venous pressure"  OR  "Blood glucose"  OR  hyperglycemia  OR  "Myocardial Infarction"  OR  "Body weight"  OR  atherosclerosis  OR  dyslipidemias  OR  lipids  OR  lipoproteins  OR  "Heart Disease”
