# Supplementary material for: The Influence of Physical Activity and Diet Mobile Apps on Cardiovascular Disease Risk Factors: Meta-Review
Source: J Med Internet Res. 2024 Oct 9;26:e51321. doi: 10.2196/51321 (PMC11499721; doi:10.2196/51321)
Supplement: Multimedia Appendix 2 [file jmir_v26i1e51321_app2.docx]

**Multimedia Appendix 2. A Summary of the Assessment of the Study Methodological Quality of the Included Meta-Analyses (N=17)**

| Author | Checklist Question | Answer | Overall Rating |
| --- | --- | --- | --- |
| Sequi-Dominguez et al. (2020) | 1. Did the research questions and inclusion criteria for the review include the components of PICO? | Yes | High |
|  | **2. Did the report of the review contain an explicit statement that the review methods were established prior to the conduct of the review and did the report justify any significant deviations from the protocol?** | Yes |  |
|  | **3. Did the review authors explain their selection of the study designs for inclusion in the review?** | No |  |
|  | 4. Did the review authors use a comprehensive literature search strategy? | Yes |  |
|  | 5. Did the review authors perform study selection in duplicate? | Yes |  |
|  | 6. Did the review authors perform data extraction in duplicate? | Yes |  |
|  | 7. Did the review authors provide a list of excluded studies and justify the exclusions? | No |  |
|  | 8. Did the review authors describe the included studies in adequate detail? | Yes |  |
|  | 9. Did the review authors use a satisfactory technique for assessing the risk of bias (RoB) in individual studies that were included in the review? | Yes |  |
|  | 10. Did the review authors report on the sources of funding for the studies included in the review? | Yes |  |
|  | 11. If meta-analysis was performed did the review authors use appropriate methods for statistical combination of results? | Yes |  |
|  | 12. If meta-analysis was performed, did the review authors assess the potential impact of RoB in individual studies on the results of the meta-analysis or other evidence synthesis? | No |  |
|  | 13. Did the review authors account for RoB in individual studies when interpreting/ discussing the results of the review? | Yes |  |
|  | 14. Did the review authors provide a satisfactory explanation for, and discussion of, any heterogeneity observed in the results of the review? | Yes |  |
|  | 15. If they performed quantitative synthesis did the review authors carry out an adequate investigation of publication bias (small study bias) and discuss its likely impact on the results of the review? | Yes |  |
|  | 16. Did the review authors report any potential sources of conflict of interest, including any funding they received for conducting the review? | Yes |  |
| Connelly et al. (2013) | 1. Did the research questions and inclusion criteria for the review include the components of PICO? | Yes | Low |
|  | **2. Did the report of the review contain an explicit statement that the review methods were established prior to the conduct of the review and did the report justify any significant deviations from the protocol?** | No |  |
|  | **3. Did the review authors explain their selection of the study designs for inclusion in the review?** | No |  |
|  | 4. Did the review authors use a comprehensive literature search strategy? | Yes |  |
|  | 5. Did the review authors perform study selection in duplicate? | Yes |  |
|  | 6. Did the review authors perform data extraction in duplicate? | Yes |  |
|  | 7. Did the review authors provide a list of excluded studies and justify the exclusions? | No |  |
|  | 8. Did the review authors describe the included studies in adequate detail? | Yes |  |
|  | 9. Did the review authors use a satisfactory technique for assessing the risk of bias (RoB) in individual studies that were included in the review? | No |  |
|  | 10. Did the review authors report on the sources of funding for the studies included in the review? | Yes |  |
|  | 11. If meta-analysis was performed did the review authors use appropriate methods for statistical combination of results? | No |  |
|  | 12. If meta-analysis was performed, did the review authors assess the potential impact of RoB in individual studies on the results of the meta-analysis or other evidence synthesis? | No |  |
|  | 13. Did the review authors account for RoB in individual studies when interpreting/ discussing the results of the review? | No |  |
|  | 14. Did the review authors provide a satisfactory explanation for, and discussion of, any heterogeneity observed in the results of the review? | No |  |
|  | 15. If they performed quantitative synthesis did the review authors carry out an adequate investigation of publication bias (small study bias) and discuss its likely impact on the results of the review? | No |  |
|  | 16. Did the review authors report any potential sources of conflict of interest, including any funding they received for conducting the review? | No |  |
| Cotterez et al. (2014) | 1. Did the research questions and inclusion criteria for the review include the components of PICO? | Yes | Low |
|  | **2. Did the report of the review contain an explicit statement that the review methods were established prior to the conduct of the review and did the report justify any significant deviations from the protocol?** | No |  |
|  | **3. Did the review authors explain their selection of the study designs for inclusion in the review?** | No |  |
|  | 4. Did the review authors use a comprehensive literature search strategy? | Yes |  |
|  | 5. Did the review authors perform study selection in duplicate? | Yes |  |
|  | 6. Did the review authors perform data extraction in duplicate? | Yes |  |
|  | 7. Did the review authors provide a list of excluded studies and justify the exclusions? | No |  |
|  | 8. Did the review authors describe the included studies in adequate detail? | Yes |  |
|  | 9. Did the review authors use a satisfactory technique for assessing the risk of bias (RoB) in individual studies that were included in the review? | No |  |
|  | 10. Did the review authors report on the sources of funding for the studies included in the review? | Yes |  |
|  | 11. If meta-analysis was performed did the review authors use appropriate methods for statistical combination of results? | No |  |
|  | 12. If meta-analysis was performed, did the review authors assess the potential impact of RoB in individual studies on the results of the meta-analysis or other evidence synthesis? | No |  |
|  | 13. Did the review authors account for RoB in individual studies when interpreting/ discussing the results of the review? | No |  |
|  | 14. Did the review authors provide a satisfactory explanation for, and discussion of, any heterogeneity observed in the results of the review? | No |  |
|  | 15. If they performed quantitative synthesis did the review authors carry out an adequate investigation of publication bias (small study bias) and discuss its likely impact on the results of the review? | No |  |
|  | 16. Did the review authors report any potential sources of conflict of interest, including any funding they received for conducting the review? | No |  |
| Cavero-Redondo et al. (2020) | 1. Did the research questions and inclusion criteria for the review include the components of PICO? | Yes | Moderate |
|  | **2. Did the report of the review contain an explicit statement that the review methods were established prior to the conduct of the review and did the report justify any significant deviations from the protocol?** | Yes |  |
|  | **3. Did the review authors explain their selection of the study designs for inclusion in the review?** | No |  |
|  | 4. Did the review authors use a comprehensive literature search strategy? | Yes |  |
|  | 5. Did the review authors perform study selection in duplicate? | No |  |
|  | 6. Did the review authors perform data extraction in duplicate? | Yes |  |
|  | 7. Did the review authors provide a list of excluded studies and justify the exclusions? | No |  |
|  | 8. Did the review authors describe the included studies in adequate detail? | Yes |  |
|  | 9. Did the review authors use a satisfactory technique for assessing the risk of bias (RoB) in individual studies that were included in the review? | Yes |  |
|  | 10. Did the review authors report on the sources of funding for the studies included in the review? | Yes |  |
|  | 11. If meta-analysis was performed did the review authors use appropriate methods for statistical combination of results? | Yes |  |
|  | 12. If meta-analysis was performed, did the review authors assess the potential impact of RoB in individual studies on the results of the meta-analysis or other evidence synthesis? | No |  |
|  | 13. Did the review authors account for RoB in individual studies when interpreting/ discussing the results of the review? | Yes |  |
|  | 14. Did the review authors provide a satisfactory explanation for, and discussion of, any heterogeneity observed in the results of the review? | Yes |  |
|  | 15. If they performed quantitative synthesis did the review authors carry out an adequate investigation of publication bias (small study bias) and discuss its likely impact on the results of the review? | Yes |  |
|  | 16. Did the review authors report any potential sources of conflict of interest, including any funding they received for conducting the review? | Yes |  |
| Veazie et al. (2018) | 1. Did the research questions and inclusion criteria for the review include the components of PICO? | Yes | Low |
|  | **2. Did the report of the review contain an explicit statement that the review methods were established prior to the conduct of the review and did the report justify any significant deviations from the protocol?** | Yes |  |
|  | **3. Did the review authors explain their selection of the study designs for inclusion in the review?** | No |  |
|  | 4. Did the review authors use a comprehensive literature search strategy? | Yes |  |
|  | 5. Did the review authors perform study selection in duplicate? | No |  |
|  | 6. Did the review authors perform data extraction in duplicate? | Yes |  |
|  | 7. Did the review authors provide a list of excluded studies and justify the exclusions? | No |  |
|  | 8. Did the review authors describe the included studies in adequate detail? | Yes |  |
|  | 9. Did the review authors use a satisfactory technique for assessing the risk of bias (RoB) in individual studies that were included in the review? | Yes |  |
|  | 10. Did the review authors report on the sources of funding for the studies included in the review? | Yes |  |
|  | 11. If meta-analysis was performed did the review authors use appropriate methods for statistical combination of results? | No |  |
|  | 12. If meta-analysis was performed, did the review authors assess the potential impact of RoB in individual studies on the results of the meta-analysis or other evidence synthesis? | No |  |
|  | 13. Did the review authors account for RoB in individual studies when interpreting/ discussing the results of the review? | No |  |
|  | 14. Did the review authors provide a satisfactory explanation for, and discussion of, any heterogeneity observed in the results of the review? | No |  |
|  | 15. If they performed quantitative synthesis did the review authors carry out an adequate investigation of publication bias (small study bias) and discuss its likely impact on the results of the review? | No |  |
|  | 16. Did the review authors report any potential sources of conflict of interest, including any funding they received for conducting the review? | Yes |  |
| Houser et al. (2019) | 1. Did the research questions and inclusion criteria for the review include the components of PICO? | Yes | Low |
|  | **2. Did the report of the review contain an explicit statement that the review methods were established prior to the conduct of the review and did the report justify any significant deviations from the protocol?** | Yes |  |
|  | **3. Did the review authors explain their selection of the study designs for inclusion in the review?** | No |  |
|  | 4. Did the review authors use a comprehensive literature search strategy? | Yes |  |
|  | 5. Did the review authors perform study selection in duplicate? | Yes |  |
|  | 6. Did the review authors perform data extraction in duplicate? | Yes |  |
|  | 7. Did the review authors provide a list of excluded studies and justify the exclusions? | No |  |
|  | 8. Did the review authors describe the included studies in adequate detail? | Yes |  |
|  | 9. Did the review authors use a satisfactory technique for assessing the risk of bias (RoB) in individual studies that were included in the review? | No |  |
|  | 10. Did the review authors report on the sources of funding for the studies included in the review? | Yes |  |
|  | 11. If meta-analysis was performed did the review authors use appropriate methods for statistical combination of results? | No |  |
|  | 12. If meta-analysis was performed, did the review authors assess the potential impact of RoB in individual studies on the results of the meta-analysis or other evidence synthesis? | No |  |
|  | 13. Did the review authors account for RoB in individual studies when interpreting/ discussing the results of the review? | No |  |
|  | 14. Did the review authors provide a satisfactory explanation for, and discussion of, any heterogeneity observed in the results of the review? | No |  |
|  | 15. If they performed quantitative synthesis did the review authors carry out an adequate investigation of publication bias (small study bias) and discuss its likely impact on the results of the review? | No |  |
|  | 16. Did the review authors report any potential sources of conflict of interest, including any funding they received for conducting the review? | No |  |
| Howland et al. (2020) | 1. Did the research questions and inclusion criteria for the review include the components of PICO? | Yes | Low |
|  | **2. Did the report of the review contain an explicit statement that the review methods were established prior to the conduct of the review and did the report justify any significant deviations from the protocol?** | Yes |  |
|  | **3. Did the review authors explain their selection of the study designs for inclusion in the review?** | No |  |
|  | 4. Did the review authors use a comprehensive literature search strategy? | Yes |  |
|  | 5. Did the review authors perform study selection in duplicate? | No |  |
|  | 6. Did the review authors perform data extraction in duplicate? | Yes |  |
|  | 7. Did the review authors provide a list of excluded studies and justify the exclusions? | No |  |
|  | 8. Did the review authors describe the included studies in adequate detail? | Yes |  |
|  | 9. Did the review authors use a satisfactory technique for assessing the risk of bias (RoB) in individual studies that were included in the review? | Yes |  |
|  | 10. Did the review authors report on the sources of funding for the studies included in the review? | Yes |  |
|  | 11. If meta-analysis was performed did the review authors use appropriate methods for statistical combination of results? | No |  |
|  | 12. If meta-analysis was performed, did the review authors assess the potential impact of RoB in individual studies on the results of the meta-analysis or other evidence synthesis? | No |  |
|  | 13. Did the review authors account for RoB in individual studies when interpreting/ discussing the results of the review? | No |  |
|  | 14. Did the review authors provide a satisfactory explanation for, and discussion of, any heterogeneity observed in the results of the review? | No |  |
|  | 15. If they performed quantitative synthesis did the review authors carry out an adequate investigation of publication bias (small study bias) and discuss its likely impact on the results of the review? | No |  |
|  | 16. Did the review authors report any potential sources of conflict of interest, including any funding they received for conducting the review? | Yes |  |
| McMahon et al. (2020) | 1. Did the research questions and inclusion criteria for the review include the components of PICO? | Yes | High |
|  | **2. Did the report of the review contain an explicit statement that the review methods were established prior to the conduct of the review and did the report justify any significant deviations from the protocol?** | Yes |  |
|  | **3. Did the review authors explain their selection of the study designs for inclusion in the review?** | No |  |
|  | 4. Did the review authors use a comprehensive literature search strategy? | Yes |  |
|  | 5. Did the review authors perform study selection in duplicate? | Yes |  |
|  | 6. Did the review authors perform data extraction in duplicate? | Yes |  |
|  | 7. Did the review authors provide a list of excluded studies and justify the exclusions? | No |  |
|  | 8. Did the review authors describe the included studies in adequate detail? | Yes |  |
|  | 9. Did the review authors use a satisfactory technique for assessing the risk of bias (RoB) in individual studies that were included in the review? | Yes |  |
|  | 10. Did the review authors report on the sources of funding for the studies included in the review? | Yes |  |
|  | 11. If meta-analysis was performed did the review authors use appropriate methods for statistical combination of results? | Yes |  |
|  | 12. If meta-analysis was performed, did the review authors assess the potential impact of RoB in individual studies on the results of the meta-analysis or other evidence synthesis? | Yes |  |
|  | 13. Did the review authors account for RoB in individual studies when interpreting/ discussing the results of the review? | Yes |  |
|  | 14. Did the review authors provide a satisfactory explanation for, and discussion of, any heterogeneity observed in the results of the review? | Yes |  |
|  | 15. If they performed quantitative synthesis did the review authors carry out an adequate investigation of publication bias (small study bias) and discuss its likely impact on the results of the review? | Yes |  |
|  | 16. Did the review authors report any potential sources of conflict of interest, including any funding they received for conducting the review? | Yes |  |
| Kuo et al. (2018) | 1. Did the research questions and inclusion criteria for the review include the components of PICO? | Yes | High |
|  | **2. Did the report of the review contain an explicit statement that the review methods were established prior to the conduct of the review and did the report justify any significant deviations from the protocol?** | Yes |  |
|  | **3. Did the review authors explain their selection of the study designs for inclusion in the review?** | No |  |
|  | 4. Did the review authors use a comprehensive literature search strategy? | Yes |  |
|  | 5. Did the review authors perform study selection in duplicate? | No |  |
|  | 6. Did the review authors perform data extraction in duplicate? | Yes |  |
|  | 7. Did the review authors provide a list of excluded studies and justify the exclusions? | No |  |
|  | 8. Did the review authors describe the included studies in adequate detail? | Yes |  |
|  | 9. Did the review authors use a satisfactory technique for assessing the risk of bias (RoB) in individual studies that were included in the review? | Yes |  |
|  | 10. Did the review authors report on the sources of funding for the studies included in the review? | Yes |  |
|  | 11. If meta-analysis was performed did the review authors use appropriate methods for statistical combination of results? | Yes |  |
|  | 12. If meta-analysis was performed, did the review authors assess the potential impact of RoB in individual studies on the results of the meta-analysis or other evidence synthesis? | Yes |  |
|  | 13. Did the review authors account for RoB in individual studies when interpreting/ discussing the results of the review? | Yes |  |
|  | 14. Did the review authors provide a satisfactory explanation for, and discussion of, any heterogeneity observed in the results of the review? | Yes |  |
|  | 15. If they performed quantitative synthesis did the review authors carry out an adequate investigation of publication bias (small study bias) and discuss its likely impact on the results of the review? | Yes |  |
|  | 16. Did the review authors report any potential sources of conflict of interest, including any funding they received for conducting the review? | Yes |  |
| Coons et al. (2012) | 1. Did the research questions and inclusion criteria for the review include the components of PICO? | Yes | Low |
|  | **2. Did the report of the review contain an explicit statement that the review methods were established prior to the conduct of the review and did the report justify any significant deviations from the protocol?** | Yes |  |
|  | **3. Did the review authors explain their selection of the study designs for inclusion in the review?** | No |  |
|  | 4. Did the review authors use a comprehensive literature search strategy? | Yes |  |
|  | 5. Did the review authors perform study selection in duplicate? | Yes |  |
|  | 6. Did the review authors perform data extraction in duplicate? | Yes |  |
|  | 7. Did the review authors provide a list of excluded studies and justify the exclusions? | No |  |
|  | 8. Did the review authors describe the included studies in adequate detail? | Yes |  |
|  | 9. Did the review authors use a satisfactory technique for assessing the risk of bias (RoB) in individual studies that were included in the review? | No |  |
|  | 10. Did the review authors report on the sources of funding for the studies included in the review? | No |  |
|  | 11. If meta-analysis was performed did the review authors use appropriate methods for statistical combination of results? | No |  |
|  | 12. If meta-analysis was performed, did the review authors assess the potential impact of RoB in individual studies on the results of the meta-analysis or other evidence synthesis? | No |  |
|  | 13. Did the review authors account for RoB in individual studies when interpreting/ discussing the results of the review? | No |  |
|  | 14. Did the review authors provide a satisfactory explanation for, and discussion of, any heterogeneity observed in the results of the review? | No |  |
|  | 15. If they performed quantitative synthesis did the review authors carry out an adequate investigation of publication bias (small study bias) and discuss its likely impact on the results of the review? | No |  |
|  | 16. Did the review authors report any potential sources of conflict of interest, including any funding they received for conducting the review? | No |  |
| Lyzwinski (2014) | 1. Did the research questions and inclusion criteria for the review include the components of PICO? | Yes | Moderate |
|  | **2. Did the report of the review contain an explicit statement that the review methods were established prior to the conduct of the review and did the report justify any significant deviations from the protocol?** | Yes |  |
|  | **3. Did the review authors explain their selection of the study designs for inclusion in the review?** | No |  |
|  | 4. Did the review authors use a comprehensive literature search strategy? | Yes |  |
|  | 5. Did the review authors perform study selection in duplicate? | No |  |
|  | 6. Did the review authors perform data extraction in duplicate? | No |  |
|  | 7. Did the review authors provide a list of excluded studies and justify the exclusions? | No |  |
|  | 8. Did the review authors describe the included studies in adequate detail? | Yes |  |
|  | 9. Did the review authors use a satisfactory technique for assessing the risk of bias (RoB) in individual studies that were included in the review? | Yes |  |
|  | 10. Did the review authors report on the sources of funding for the studies included in the review? | No |  |
|  | 11. If meta-analysis was performed did the review authors use appropriate methods for statistical combination of results? | Yes |  |
|  | 12. If meta-analysis was performed, did the review authors assess the potential impact of RoB in individual studies on the results of the meta-analysis or other evidence synthesis? | Yes |  |
|  | 13. Did the review authors account for RoB in individual studies when interpreting/ discussing the results of the review? | Yes |  |
|  | 14. Did the review authors provide a satisfactory explanation for, and discussion of, any heterogeneity observed in the results of the review? | Yes |  |
|  | 15. If they performed quantitative synthesis did the review authors carry out an adequate investigation of publication bias (small study bias) and discuss its likely impact on the results of the review? | Yes |  |
|  | 16. Did the review authors report any potential sources of conflict of interest, including any funding they received for conducting the review? | Yes |  |
| Sherrington et al. (2016) | 1. Did the research questions and inclusion criteria for the review include the components of PICO? | Yes | High |
|  | **2. Did the report of the review contain an explicit statement that the review methods were established prior to the conduct of the review and did the report justify any significant deviations from the protocol?** | Yes |  |
|  | **3. Did the review authors explain their selection of the study designs for inclusion in the review?** | No |  |
|  | 4. Did the review authors use a comprehensive literature search strategy? | Yes |  |
|  | 5. Did the review authors perform study selection in duplicate? | Yes |  |
|  | 6. Did the review authors perform data extraction in duplicate? | Yes |  |
|  | 7. Did the review authors provide a list of excluded studies and justify the exclusions? | No |  |
|  | 8. Did the review authors describe the included studies in adequate detail? | Yes |  |
|  | 9. Did the review authors use a satisfactory technique for assessing the risk of bias (RoB) in individual studies that were included in the review? | Yes |  |
|  | 10. Did the review authors report on the sources of funding for the studies included in the review? | Yes |  |
|  | 11. If meta-analysis was performed did the review authors use appropriate methods for statistical combination of results? | Yes |  |
|  | 12. If meta-analysis was performed, did the review authors assess the potential impact of RoB in individual studies on the results of the meta-analysis or other evidence synthesis? | Yes |  |
|  | 13. Did the review authors account for RoB in individual studies when interpreting/ discussing the results of the review? | Yes |  |
|  | 14. Did the review authors provide a satisfactory explanation for, and discussion of, any heterogeneity observed in the results of the review? | Yes |  |
|  | 15. If they performed quantitative synthesis did the review authors carry out an adequate investigation of publication bias (small study bias) and discuss its likely impact on the results of the review? | No |  |
|  | 16. Did the review authors report any potential sources of conflict of interest, including any funding they received for conducting the review? | Yes |  |
| Cotie et al. (2018) | 1. Did the research questions and inclusion criteria for the review include the components of PICO? | Yes | High |
|  | **2. Did the report of the review contain an explicit statement that the review methods were established prior to the conduct of the review and did the report justify any significant deviations from the protocol?** | Yes |  |
|  | **3. Did the review authors explain their selection of the study designs for inclusion in the review?** | No |  |
|  | 4. Did the review authors use a comprehensive literature search strategy? | Yes |  |
|  | 5. Did the review authors perform study selection in duplicate? | Yes |  |
|  | 6. Did the review authors perform data extraction in duplicate? | Yes |  |
|  | 7. Did the review authors provide a list of excluded studies and justify the exclusions? | No |  |
|  | 8. Did the review authors describe the included studies in adequate detail? | Yes |  |
|  | 9. Did the review authors use a satisfactory technique for assessing the risk of bias (RoB) in individual studies that were included in the review? | Yes |  |
|  | 10. Did the review authors report on the sources of funding for the studies included in the review? | Yes |  |
|  | 11. If meta-analysis was performed did the review authors use appropriate methods for statistical combination of results? | Yes |  |
|  | 12. If meta-analysis was performed, did the review authors assess the potential impact of RoB in individual studies on the results of the meta-analysis or other evidence synthesis? | Yes |  |
|  | 13. Did the review authors account for RoB in individual studies when interpreting/ discussing the results of the review? | Yes |  |
|  | 14. Did the review authors provide a satisfactory explanation for, and discussion of, any heterogeneity observed in the results of the review? | Yes |  |
|  | 15. If they performed quantitative synthesis did the review authors carry out an adequate investigation of publication bias (small study bias) and discuss its likely impact on the results of the review? | Yes |  |
|  | 16. Did the review authors report any potential sources of conflict of interest, including any funding they received for conducting the review? | Yes |  |
| Puig et al. (2019) | 1. Did the research questions and inclusion criteria for the review include the components of PICO? | Yes | Moderate |
|  | **2. Did the report of the review contain an explicit statement that the review methods were established prior to the conduct of the review and did the report justify any significant deviations from the protocol?** | Yes |  |
|  | **3. Did the review authors explain their selection of the study designs for inclusion in the review?** | No |  |
|  | 4. Did the review authors use a comprehensive literature search strategy? | Yes |  |
|  | 5. Did the review authors perform study selection in duplicate? | Yes |  |
|  | 6. Did the review authors perform data extraction in duplicate? | Yes |  |
|  | 7. Did the review authors provide a list of excluded studies and justify the exclusions? | No |  |
|  | 8. Did the review authors describe the included studies in adequate detail? | Yes |  |
|  | 9. Did the review authors use a satisfactory technique for assessing the risk of bias (RoB) in individual studies that were included in the review? | Yes |  |
|  | 10. Did the review authors report on the sources of funding for the studies included in the review? | Yes |  |
|  | 11. If meta-analysis was performed did the review authors use appropriate methods for statistical combination of results? | No |  |
|  | 12. If meta-analysis was performed, did the review authors assess the potential impact of RoB in individual studies on the results of the meta-analysis or other evidence synthesis? | No |  |
|  | 13. Did the review authors account for RoB in individual studies when interpreting/ discussing the results of the review? | No |  |
|  | 14. Did the review authors provide a satisfactory explanation for, and discussion of, any heterogeneity observed in the results of the review? | Yes |  |
|  | 15. If they performed quantitative synthesis did the review authors carry out an adequate investigation of publication bias (small study bias) and discuss its likely impact on the results of the review? | No |  |
|  | 16. Did the review authors report any potential sources of conflict of interest, including any funding they received for conducting the review? | Yes |  |
| Buckingham et al. (2018) | 1. Did the research questions and inclusion criteria for the review include the components of PICO? | Yes | Moderate |
|  | **2. Did the report of the review contain an explicit statement that the review methods were established prior to the conduct of the review and did the report justify any significant deviations from the protocol?** | Yes |  |
|  | **3. Did the review authors explain their selection of the study designs for inclusion in the review?** | No |  |
|  | 4. Did the review authors use a comprehensive literature search strategy? | Yes |  |
|  | 5. Did the review authors perform study selection in duplicate? | Yes |  |
|  | 6. Did the review authors perform data extraction in duplicate? | Yes |  |
|  | 7. Did the review authors provide a list of excluded studies and justify the exclusions? | No |  |
|  | 8. Did the review authors describe the included studies in adequate detail? | Yes |  |
|  | 9. Did the review authors use a satisfactory technique for assessing the risk of bias (RoB) in individual studies that were included in the review? | Yes |  |
|  | 10. Did the review authors report on the sources of funding for the studies included in the review? | Yes |  |
|  | 11. If meta-analysis was performed did the review authors use appropriate methods for statistical combination of results? | No |  |
|  | 12. If meta-analysis was performed, did the review authors assess the potential impact of RoB in individual studies on the results of the meta-analysis or other evidence synthesis? | No |  |
|  | 13. Did the review authors account for RoB in individual studies when interpreting/ discussing the results of the review? | No |  |
|  | 14. Did the review authors provide a satisfactory explanation for, and discussion of, any heterogeneity observed in the results of the review? | Yes |  |
|  | 15. If they performed quantitative synthesis did the review authors carry out an adequate investigation of publication bias (small study bias) and discuss its likely impact on the results of the review? | No |  |
|  | 16. Did the review authors report any potential sources of conflict of interest, including any funding they received for conducting the review? | Yes |  |
| Kim et al. (2020) | 1. Did the research questions and inclusion criteria for the review include the components of PICO? | Yes | High |
|  | **2. Did the report of the review contain an explicit statement that the review methods were established prior to the conduct of the review and did the report justify any significant deviations from the protocol?** | Yes |  |
|  | **3. Did the review authors explain their selection of the study designs for inclusion in the review?** | No |  |
|  | 4. Did the review authors use a comprehensive literature search strategy? | Yes |  |
|  | 5. Did the review authors perform study selection in duplicate? | Yes |  |
|  | 6. Did the review authors perform data extraction in duplicate? | Yes |  |
|  | 7. Did the review authors provide a list of excluded studies and justify the exclusions? | No |  |
|  | 8. Did the review authors describe the included studies in adequate detail? | Yes |  |
|  | 9. Did the review authors use a satisfactory technique for assessing the risk of bias (RoB) in individual studies that were included in the review? | Yes |  |
|  | 10. Did the review authors report on the sources of funding for the studies included in the review? | Yes |  |
|  | 11. If meta-analysis was performed did the review authors use appropriate methods for statistical combination of results? | Yes |  |
|  | 12. If meta-analysis was performed, did the review authors assess the potential impact of RoB in individual studies on the results of the meta-analysis or other evidence synthesis? | Yes |  |
|  | 13. Did the review authors account for RoB in individual studies when interpreting/ discussing the results of the review? | Yes |  |
|  | 14. Did the review authors provide a satisfactory explanation for, and discussion of, any heterogeneity observed in the results of the review? | Yes |  |
|  | 15. If they performed quantitative synthesis did the review authors carry out an adequate investigation of publication bias (small study bias) and discuss its likely impact on the results of the review? | No |  |
|  | 16. Did the review authors report any potential sources of conflict of interest, including any funding they received for conducting the review? | Yes |  |
| Daryabeygi-Khotbehsara et al. (2021) | 1. Did the research questions and inclusion criteria for the review include the components of PICO? | Yes | Moderate |
|  | **2. Did the report of the review contain an explicit statement that the review methods were established prior to the conduct of the review and did the report justify any significant deviations from the protocol?** | Yes |  |
|  | **3. Did the review authors explain their selection of the study designs for inclusion in the review?** | No |  |
|  | 4. Did the review authors use a comprehensive literature search strategy? | Yes |  |
|  | 5. Did the review authors perform study selection in duplicate? | Yes |  |
|  | 6. Did the review authors perform data extraction in duplicate? | Yes |  |
|  | 7. Did the review authors provide a list of excluded studies and justify the exclusions? | No |  |
|  | 8. Did the review authors describe the included studies in adequate detail? | Yes |  |
|  | 9. Did the review authors use a satisfactory technique for assessing the risk of bias (RoB) in individual studies that were included in the review? | Yes |  |
|  | 10. Did the review authors report on the sources of funding for the studies included in the review? | Yes |  |
|  | 11. If meta-analysis was performed did the review authors use appropriate methods for statistical combination of results? | No |  |
|  | 12. If meta-analysis was performed, did the review authors assess the potential impact of RoB in individual studies on the results of the meta-analysis or other evidence synthesis? | No |  |
|  | 13. Did the review authors account for RoB in individual studies when interpreting/ discussing the results of the review? | Yes |  |
|  | 14. Did the review authors provide a satisfactory explanation for, and discussion of, any heterogeneity observed in the results of the review? | Yes |  |
|  | 15. If they performed quantitative synthesis did the review authors carry out an adequate investigation of publication bias (small study bias) and discuss its likely impact on the results of the review? | No |  |
|  | 16. Did the review authors report any potential sources of conflict of interest, including any funding they received for conducting the review? | Yes |  |
