# Supplementary material for: The Influence of Physical Activity and Diet Mobile Apps on Cardiovascular Disease Risk Factors: Meta-Review
Source: J Med Internet Res. 2024 Oct 9;26:e51321. doi: 10.2196/51321 (PMC11499721; doi:10.2196/51321)
Supplement: Multimedia Appendix 4 [file jmir_v26i1e51321_app4.docx]

**Multimedia Appendix 3**. **A Summary of the Sample, Study, and Intervention Characteristics of the included RCTs within the Qualifying Systematic Reviews and Meta-Analyses (N=17)**

| Author, Date | Sample and Study Characteristics | Intervention Characteristics | Measurements/ Outcomes | PA or PA and Diet Study | Additional Comments |
| --- | --- | --- | --- | --- | --- |
| Sequi-Dominguez et al., 2020 | -Health Outcome: Metabolic Syndrome  -Age: The mean age varied between 38.4 and 59.7 years.  -Gender: 51.7% females, although two studies included men only.  -Participants: The sample size of the included studies ranged from 12 to 421 participants.  -Location: Spain  -Study Design: systematic review/ meta-analysis  -Population: Individuals with Metabolic Syndrome | -Intervention Description: The interventions were mainly based on physical activity and lifestyle recommendations, with personalization in some cases.  -Intervention Duration Range: The duration of interventions ranged from 8 to 48 weeks.  -Platform Use: Website, videoconferencing, or an app.  -Control: No information provided. | -Primary outcomes: BMI, waist circumference, systolic blood pressure, diastolic blood pressure, fasting plasma glucose, HbA1c, total cholesterol, high-density lipoprotein cholesterol, low-density lipoprotein cholesterol, and triglycerides. | PA and Diet | -9 Studies  -Study Description: Of the included studies, five were RCTs and four were single-arm pre-post studies. Studies were published between 2013 and 2018 and conducted in four different countries (two in Canada, one in Germany, three in the Republic of Korea, and three in the United States. |
| Connelly et al., 2013 | -Health Outcome: Type 2 Diabetes  -Age: 18 years or older  -Gender: No information provided.  -Participants: Sample size ranged from 26 to 958  participants.  -Location: United Kingdom  -Study Design: systematic review  -Population: Individuals with Type 2 Diabetes | -Intervention Description: An intervention where technology was the main method of delivery and an intervention promoting diabetes management through physical activity.  -Intervention Duration Range: Interventions lasted between  6 weeks and 1 year, with four having a follow-up period of  between 6 and 18 months.  -Platform Use: Web-based, mobile phone, CD-ROM, computer based.  -Control: No information provided. | -Primary outcomes: Physical Activity | PA and Diet | -15 Studies  -Study Description: Eleven studies were conducted in the USA, two in Canada, one in South Korea and one in Iran. |
| Cotterez et al., 2013 | -Health Outcome: Type 2 Diabetes  -Age: 18 years or older  -Gender: No information provided.  -Participants: Sample size ranged from 35 to 761 participants.  -Location: USA  -Study Design: systematic review  -Population: Individuals with Type 2 Diabetes | -Intervention Description: Each study created a web-based intervention designed to promote diabetes education and some health behavior change. The most targeted behaviors included being active (8/9), healthy eating (6/9), and glucose monitoring (5/9).  -Intervention Duration Range: The duration of interventions ranged from 6 weeks to 18 months.  -Platform Use: Web-based  -Control: No information provided. | -Primary outcomes: Physical Activity, HbA1c | PA and Diet | -9 Studies  -Study Description: Eight of the studies reviewed were randomized controlled trials with patients with type 2 diabetes (5 small trials < 100 participants; 3 larger trials > 300 participants) and one was quasi-experimental with a control group (n=98). |
| Cavero-Redondo et al., 2020 | -Health Outcome: Obesity  -Age: The mean age varied between 20.5 and 59.8 years.  -Gender: No information provided.  -Participants: Sample sizes ranged from 11 to 131 participants in the lifestyle mHealth self-monitoring intervention groups and from six to 133 participants in the control groups.  -Location: Spain  -Study Design: systematic review/MA  -Population: General Population | -Intervention Description: Studies comparing the effect of lifestyle (diet and physical activity) mHealth self-monitoring (i.e., personal digital assistants (PDAs), smartphones or web-based)  -Intervention Duration Range: The length of the interventions ranged from one to 24 months.  -Platform Use: PDAs, smartphones and web-based approaches. Additionally, 16 studies specified the application used for lifestyle mHealth self-monitoring: Lose It!, TXT2BFiT, Dietmate Pro, My Meal Mate, MyFitnessPal, Be Positive Be Healthy, SmartLoss, CalorieKing and Fitbit.  -Control: Comparator groups included usual care, paper records and wait-lists. | -Primary outcomes: Weight change (BMI, waist circumference) | PA and Diet | -20 Studies  -Study Description: Of the studies included, 12 were conducted in the United States, two in the United Kingdom, three in Australia, one in New Zealand, one in South Korea and one in Finland. Reports were published between 2007 and 2019, and they included studies using the following experimental designs: 17 RCTs and three non-RCTs. |
| Veazie et al., 2018 | -Health Outcome: Diabetes  -Age: Participants ranged in mean age from 33 to 40 years old in type 1 diabetes studies. Participants ranged in mean age from 48 to 55 years old in type 2 diabetes studies.  -Gender: No information provided.  -Participants: Sample size ranged from 30 to 180 participants for type 1 diabetes studies. Sample size ranged from 30 to 163 participants for type 2 diabetes studies.  -Location: USA  -Study Design: systematic review  -Population: Individuals with Type 1 or 2 Diabetes | -Intervention Description: Six apps (Glucose Buddy, Diabetes Manager, Diabetes Diary, Dbees, Diabetes Interactive Diary, and Diabeo Telesage) were identified for type 1 and five apps (BlueStar Diabetes [BlueStar], WellTang, NextJ Connected Wellness Platform Health Coach + [NextJ], Gather Health, and mDiab) were for type 2 diabetes. Common features of apps for type 1 and 2 diabetes included the ability to track health data such as blood glucose, carbohydrates/food, prescriptions, and exercise; patient feedback such as reminders to take medication or measure blood glucose; and diabetes education.  -Intervention Duration Range: Study duration ranged from 8 weeks to 6 months in type 1 diabetes studies. Study duration ranged from 2 to 12 months in type 2 diabetes studies.  -Platform Use: Commercially available website, program, or application delivered through a mobile device.  -Control: Usual care or other mobile or nonmobile program for diabetes self-management. | -Primary outcomes: Diabetes related outcomes | PA and Diet | -15 Studies  -Study Description: Eight articles from seven studies were identified for type 1 diabetes evaluating six commercially available mobile applications for type 1 diabetes. Among the eight articles identified, seven were RCTs and one was a subgroup analysis of an included RCT. Seven articles of six studies were identified evaluating five commercially available apps for type 2 diabetes. Of the seven publications, six were RCTs and one was a subgroup analysis of an included RCT. |
| Houser et al., 2019 | -Health Outcome: Obesity  -Age: No information provided.  -Gender: No information provided.  -Participants: The study sample sizes ranged from 20 to nearly 2,500 participants.  -Location: USA  -Study Design: systematic review  -Population: General Population | -Intervention Description: The most common type of technology used for the management of obesity was mobile devices, such as mobile phone applications and text messages (11 studies; 48 percent). Five studies (22 percent) used telehealth and telemedicine components, such as telephone counseling, videoconferencing, and interactive telemonitoring; six studies (26 percent) relied on eHealth in the form of websites and internet-based programs; and one study (4.3 percent) monitored weight loss using a wearable device.  -Intervention Duration Range: No information provided  -Platform Use: Mobile health, eHealth, and telehealth/telemedicine are among the most popular technologies used.  -Control: No information provided. | -Primary outcomes: Study outcome measurements include association between technology use and weight loss, changes in body mass index, dietary habits, and physical activities. | PA | -23 Studies  -Study Description: No additional information provided. |
| Howland et al., 2020 | -Health Outcome: Diabetes  -Age: The mean age of participants ranged from 46 to 66.7 years.  -Gender: In seven studies, there were more females  than males enrolled in the study  -Participants: Sample sizes ranged from 20 to 1229 participants.  -Location: USA  -Study Design: systematic review  -Population: Individuals with Type 2 Diabetes. | -Intervention Description: PA and/or SB intervention delivered via telehealth strategy. The most common intervention feature was education about diabetes self‐management and/or PA. A logbook for self‐monitoring was included in 11 studies, planning or goal setting was addressed in 9 studies, and tailored feedback was included in 9 studies. Additional intervention components included videos, quizzes, barrier identification, local activities, peer support, reminder messages, professional facilitators, and peer facilitators.  -Intervention Duration Range: The duration of interventions ranged from 2 to 6 months.  -Platform Use: Ten studies delivered the intervention in only a web‐based format, two studies used a mobile app for intervention delivery, three studies used a combined web and mobile device delivered intervention Two studies supplemented a web‐based intervention delivery format with telephone calls.  -Control: No information provided. | -Primary outcomes: Physical Activity | PA | -17 Studies  -Study Description: Thirteen articles were randomized controlled trials. Five randomized controlled trials compared two levels of the intervention with a control group. Two studies used a quasi‐experimental design. Two studies used a mixed method  design, with a qualitative component used for intervention development, with one including a randomized controlled trial and one a quasi‐experimental design component as pilot studies. |
| McMahon et al., 2021 | -Health Outcome: Multiple CVD risk factors  -Age: 44.2 ± 3.8  -Gender: All male  -Participants: Study sample sizes ranged from 39 to 441 at the point of randomization.  -Location: United Kingdom  -Study Design: systematic review/ meta-analysis  -Population: Men 18 years or older | -Intervention Description: eHealth interventions targeting a minimum of two major CVD-related risk factors (e.g., weight, physical activity, diet)  -Intervention Duration Range: Intervention durations ranged between one and 12-months  -Platform Use: No information provided  -Control: The choice of comparison groups in the nine trials varied: four used a wait-list control group, four used a comparison group which received printed resources related to the online intervention, and one used a ‘true control’ group, i.e., receiving no intervention or materials. | -Primary outcomes: Weight, PA, diet/nutrition, and related biomarkers/clinical measures: blood pressure, cholesterol, glucose, and HbA1c. | PA and Diet | -9 Studies  -Study Description: Of the nine trials reviewed, five were conducted in  Australia, three in the USA and one in Japan. Six trials targeted  men who were overweight or obese, two middle aged, and one in the military |
| Kuo et al., 2018 | -Health Outcome: Metabolic Syndrome  -Age: No information provided.  -Gender: No information provided.  -Participants: Sample sizes ranged from 30 to 786 subjects.  -Location: Taiwan  -Study Design: systematic review/meta-analysis  -Population: Adults with metabolic diseases, such as metabolic syndrome, diabetes, hypertension, obesity and hyperlipidemia | -Intervention Description: Examination of IESMIs, defined as those where participants adopted strategies of self-awareness, goal setting, action planning, problem-solving and reflection to self-manage their diseases through Internet-based communication networks (e.g., computers and mobile phones)  -Intervention Duration Range: Intervention durations ranged from 2 weeks to 12 months and post-test times ranged from 8 weeks to 12 months.  -Platform Use: Internet-based communication networks (e.g., computers and mobile phones).  -Control: Comparison with usual care, wait-list control groups, or non–Internet-based interventions. | -Primary outcomes: Evaluation of behavioral (exercise and dietary habits) and physiological (HbA1c level and weight) | PA and Diet | -21 Studies  -Study Description: Sixteen of 21 studies involved subjects with diabetes, four involved obese subjects and one involved subject with hypertension. Eleven studies enrolled subjects in the United States. |
| Coons et al., 2012 | -Health Outcome: Obesity  -Age: No information provided.  -Gender: No information provided.  -Participants: Sample sizes ranged from 55 to 481 participants.  -Location: USA  -Study Design: systematic review  -Population: Overweight and obese adults. | -Intervention Description: Ten trials tested Internet interventions and 3 trials tested handheld devices (e.g., PDAs, armband with tri-axial accelerometer). Regardless of the technology platform, intervention components included 1) education about diet, physical activity, and weight management; 2) self-monitoring of diet, physical activity, and weight parameters (e.g., weight, BMI, waist circumference); and 3) goal setting for diet and physical activity. Furthermore, several trials included elements of motivational enhancement, and some provided social support through e-mail or Internet chat room contact with both coaches and peers  -Intervention Duration Range: Intervention durations ranged from 3 to 9 months.  -Platform Use: Internet and handheld devices  -Control: No information provided. | -Primary outcomes: Weight loss outcome variable(s) (e.g., weight, weight change, body mass index (BMI), waist circumference) | PA and Diet | -13 Studies  -Study Description: No additional information provided. |
| Lyzwinski, 2014 | -Health Outcome: Obesity  -Age: Mean subject age ranged from 20.4 years to 57 years of age.  -Gender: Most of the studies consisted of primarily female subjects ranging from 64% to 100% of the sample.  -Participants: Sample size varied from 52 subjects to 210 subjects.  -Location: United Kingdom  -Study Design: systematic review/ meta-analysis  -Population: Overweight and obese adults. | -Intervention Description: No additional information provided.  -Intervention Duration Range: Trial length ranged from 4 weeks to 2 years.  -Platform Use: A total of 8 out of the 12 interventions had a mobile phone as an intervention medium. Three studies did not utilize a mobile phone component. Two of these studies utilized a podcasting component employing an Mp3 player or an iPod as an intervention medium. The remaining two studies used a PDA for weight loss, of which one study combined a PDA with a mobile phone for calling purposes only.  -Control: No information provided. | -Primary outcomes: Weight loss outcome variable(s) (e.g., weight, weight change, body mass index (BMI), waist circumference) | PA and Diet | -17 Studies  -Study Description: Study locations included the UK, USA, Finland, and Australia. A total of seven studies were undertaken in the USA, three studies in the UK, one study in Finland, and one study was undertaken in Australia. |
| Sherrington et al., 2016 | -Health Outcome: Obesity  -Age: No information provided.  -Gender: 51.2% of the participant population was female.  -Participants: Sample size ranged from 65 to 1386 participants.  -Location: United Kingdom  -Study Design: systematic review/ meta-analysis  -Population: Overweight and obese adults. | -Intervention Description: All 12 studies used personalized feedback to target information received on participant's weight loss progress or individual behavior change, such as diet or physical activity level. Participant access to the internet‐delivered personalized feedback was via the website (four studies) or via emails containing the feedback (six studies), with two studies remaining unclear in how it was administered. Targeting diet and/or physical activity for weight loss, delivered at least in part via the internet, incorporating any form of individualized feedback to the participants either human‐delivered (provided by a health care professional or researcher) or computer‐generated personalized feedback (using algorithms that sent pre‐programmed responses based on participant input or choices) delivered via web‐based messages or email  -Intervention Duration Range: The length of the active interventions ranged from 3 to 24 months.  -Platform Use: Across the 12 studies, 8 incorporated human‐delivered internet feedback and 5 provided computer‐generated internet feedback.  -Control: The studies varied in terms of the features of control/comparison arms. | -Primary outcomes: Body weight change | PA and Diet | -12 Studies  -Study Description: All studies took place between 2001 and 2012. The majority (seven) were conducted in the USA, three in Australia, one in the Netherlands and one in the UK. |
| Cotie et al., 2018 | -Health Outcome: Physical Inactivity and Obesity  -Age: Mean age ranging from 18 to 65 years.  -Gender: All samples in these studies had >80% females.  -Participants: A total of 8,354 women. Sample sizes ranged from three to 855.  -Location: Canada  -Study Design: systematic review/ meta-analysis  -Population: Working aged women | -Intervention Description: Some studies used technology to deliver prompts such as reminders to exercise, track movement or encouragement to continue. More specifically, studies sent e-mails, regular text messages and/or made regular telephone calls.  -Intervention Duration Range: No information provided.  -Platform Use: Many utilized the internet, with interventions including informational websites; websites with PA tracking tools and online discussion forums for social support.  -Control: No restrictions were placed on the type of control groups (e.g., no PA intervention, low-intensity PA and print material). | -Primary outcomes: Physical Activity | PA | -20 Studies  -Study Description: Studies included in the review were published between 2000 and 2016 and conducted in four high income OECD countries (USA = 44, Australia = 9, Canada = 3, United Kingdom = 4). Forty-one RCTs; 13 pre-posts; 2 quasi-experimental; 1 randomized (no control); 1 ABA withdrawal design; 1 within-subjects trial and 1 prospective trial examined changes in MVPA levels following eHealth interventions. All papers were published in English. |
| Puig et al., 2019 | -Health Outcome: Obesity  -Age: No information provided.  -Gender: Most studies had a majority of adult women; in 6, all participants were women.  -Participants: The number of participants ranged from 10 to 1012, but most studies covered less than 100 people.  -Location: Spain  -Study Design: systematic review  -Population: General Population | -Intervention Description: Regarding the specificities of mHealth interventions, only 39% (10 out of 28) focused on a specific stand-alone app, with the majority addressing multicomponent interventions—including armband sensors, pedometers, wireless scales, and other monitoring devices, or websites—for weight management, intended to increase PA, reduce sedentary habits, and/or improve dietary patterns. The most common elements included in the trials were the receiving of feedback messages, setting of goals, and self-monitoring. These feedback messages could be personalized reminders, recommendations based on the self-monitoring, standard counseling or health coach counseling through the app, and/or a more synchronic intervention.  -Intervention Duration Range: Apart from one 24-month trial, the studies were conducted over short periods of time, ranging from 3 weeks to 6 months.  -Platform Use: mHealth interventions, mobile apps.  -Control: No information provided. | -Primary outcomes: Reduction of weight and body mass index (BMI) as well as waist and hip circumferences; changes in dietary habits, PA, and blood pressure | PA and Diet | -28 Studies  -Study Description: 13 (46%) were randomized control trials, 11 were single-arm studies (39%), 3 were nonrandomized controlled trials (11%), and 1 study was a cluster randomized trial (4%). In 11 studies, the apps were used as stand-alone interventions, the others were multicomponent studies that included other tools for support such as sensors or websites. The countries where the studies were carried out were the United States, Australia, Korea, the United Kingdom, Belgium, Spain, The Netherlands, China, and Israel. |
| Buckingham et al., 2019 | -Health Outcome: Physical Inactivity  -Age: 18 years or older.  -Gender: Of the 25 studies, 16 had a markedly higher proportion of female (≥60%) than male participants.  -Participants: Sample size ranged from 20 to 69,219 participants.  -Location: United Kingdom  -Study Design: systematic review  -Population: Workplace population | -Intervention Description: The main mHealth tools used were wearable activity monitors or trackers (n = 11), smartphone apps (n = 6), or a combination of the two (n = 8). Some studies had additional mHealth and technology intervention components, including motivational or persuasive text messaging or e-mails, computer software or websites linked to the activity monitor, and dedicated social media groups. Eleven studies assessed mHealth as a standalone intervention, whereas 14 studies used mHealth in the context of a multi-component workplace health or PA program. Among the multi-component programs in particular, interventions were diverse and additional components included educational materials on health and PA, managerial support, financial incentives or rewards, shared active workstations, online or telephone counselling, personalized feedback on activity, wellness education delivered in the workplace, group-based action planning.  -Intervention Duration Range: Intervention duration ranged from 6 weeks to 12 months.  -Platform Use: No information provided.  -Control: A control or comparator group was present in 14 of the 25 studies. Of these, six could not be classed as a ‘true’ control group as the participants received at least a partial mHealth intervention, and another three studies supplied controls with wearable activity monitors for data collection. | -Primary outcomes: Physical Activity | PA | -25 Studies  -Study Description: Eleven studies were conducted in the USA, five in Australia, two in Canada, two in the Netherlands, one in Belgium, Singapore, Finland, Norway, and one in multiple countries. The most common study designs were individual RCTs (n = 10) and pre-post prospective cohort studies (n = 10). |
| Kim et al., 2019 | -Health Outcome: Physical Inactivity and Obesity  -Age: No information provided.  -Gender: No information provided.  -Participants: A total of 1830 people were included in the meta-analysis. The number of subjects ranged from 51 to 1107.  -Location: Korea  -Study Design: systematic review/ meta-analysis  -Population: Young Adults | -Intervention Description: The intervention programs consisted of lifestyle intervention, education and coaching to promote physical activity, dietary counseling, education related to health behavior, and feedback which were provided 5–24 times over two to six months. By using smartphone applications as an intervention strategy, the subjects could directly record their health and nutrition status and receive feedback. Text messages or emails tailored by the information a subject provided were sent, or education and coaching calls personalized depending upon their health and nutrition status were offered. In some cases, smartphone apps and additional web-based education were provided, and devices were offered to check lecture-style education and steps walked. In the study that provided a step count device, the comparison group was also offered the device but did not give feedback on the results of their steps.  -Intervention Duration Range: Intervention duration ranged from 2 to 6 months.  -Platform Use: Internet and app  -Control: No information provided. | -Primary outcomes: Physical activity and obesity related outcomes | PA and Diet | -5 Studies  -Study Description: Four papers with a randomized controlled trial (RCT) research design were evaluated and one with a quasi-experimental design. The publication years were from 2006 to 2019. |
| Daryabeygi-Khotbehsara et al., 2021 | -Health Outcome: Physical Inactivity  -Age: 18 years or older  -Gender: Participants were predominantly women in all studies except one.  -Participants: Sample sizes ranged from 10 to 104 participants in the intervention evaluation studies and 60 in the development study.  -Location: Australia  -Study Design: systematic review  -Population: General Population | -Intervention Description: All studies promoted PA, whereas 4 studies also involved interventions for reducing SB. The most common behavior change technique used across all studies was goal setting. In terms of PA, 3 studies included daily goal setting to achieve PA targets, whereas 1 study promoted weekly goal setting. In a study, weekly step goals were initially established and then broken down into daily short-term goals. Only 1 study set goals for SB. Monitoring and feedback on behavior was another widely used behavior change technique. In terms of the type of intervention, 2 studies used push notification messages, 3 used push notifications to present step goals or minutes of activity goals (e.g., walking), 2 had in-app suggestions selected from frequent and infrequent past activities, and 1 used text messages.  -Intervention Duration Range: The duration of the studies ranged from 3 weeks to 6 months.  -Platform Use: Mobile apps  -Control: No information provided. | -Primary outcomes: Physical activity | PA | -11 Studies  -Study Description: A total of 5 studies were conducted in the United States, 1 in Italy, 1 in New Zealand, and 1 in the Netherlands. Of these, 3 studies used pre-post intervention designs, 2 were RCTs, 1 was a 3-arm quasi-experimental study, 1 was a single-group micro randomized trials, and 1 was development study. |
